# Peptoids and polyamines going sweet: Modular synthesis of glycosylated peptoids and polyamines using click chemistry

**DOI:** 10.3762/bjoc.9.7

**Published:** 2013-01-10

**Authors:** Daniel Fürniss, Timo Mack, Frank Hahn, Sidonie B L Vollrath, Katarzyna Koroniak, Ute Schepers, Stefan Bräse

**Affiliations:** 1 Institute of Organic Chemistry, Karlsruhe Institute of Technology (KIT), Fritz-Haber-Weg 6, 76131 Karlsruhe, Germany; 2 Kekulé Institute of Organic Chemistry and Biochemistry, Rheinische Friedrich Wilhelms University of Bonn, Germany; 3 Institute of Toxicology and Genetics, Karlsruhe Institute of Technology (KIT), Hermann-von-Helmholtz-Platz 1, 76344 Eggenstein-Leopoldshafen, Germany

**Keywords:** click chemistry, glycans, peptoids, polyalkynes, polyamines, solid-phase chemistry

## Abstract

Sugar moieties are present in a wide range of bioactive molecules. Thus, having versatile and fast methods for the decoration of biomimetic molecules with sugars is of fundamental importance. The glycosylation of peptoids and polyamines as examples of such biomimetic molecules is reported here. The method uses Cu-catalyzed azide alkyne cycloaddition to promote the reaction of azidosugars with either polyamines or peptoids. In addition, functionalized nucleic acids were attached to polyamines via the same route. Based on a modular solid-phase synthesis of peralkynylated peptoids with up to six alkyne groups, the latter were modified with azidosugar building blocks by using copper-catalyzed azide alkyne cycloadditions. In addition, the up-scaling of some particular azide-modified sugars is described.

## Introduction

To date, oligosaccharides have gained more and more interest as potential drugs in the treatment of a variety of diseases. However, the rendering of nucleic acids and oligosaccharides as therapeutically active substances often requires a derivatization or a chemical coupling reaction that permits the selective and simple formation of covalent adducts. Some modifications permit the attachment to other molecules through a variety of functional groups, such as amines (–NH_2_) and carboxylic acids (–COOH) resulting in peptide bonds, thiols (–SH) resulting in disulfides, thioethers or thioesters, aldehydes (–CHO) and hydroxy (–OH) groups. Nonetheless, coupling to these groups often requires a laborious protection of other reactive functional groups as they can compete in the coupling step. A matched pair of groups, which are selective in reacting with each other while being unreactive with other functional groups in the molecule would, therefore, be highly useful in the preparation of functional structures. Likewise, the coupling reaction should be permitted in hydrophilic solvents such as water or DMSO, since both unprotected nucleic acids and oligosaccharides, as well as many other biomacromolecules, prefer a hydrophilic reaction environment.

With the advent of mild and biocompatible conjugation methods such as the Staudinger ligation [1] or the copper-catalyzed alkyne azide cycloaddition (CuAAC) [2–3], a large number of versatile and functional bioconjugates are accessible for various applications in chemical biology [4].

To date, many therapeutically active molecules are synthetic derivatives of biomacromolecules that have to be soluble in hydrophilic environments to be taken up in vivo or in cell culture. Common solubilizers that enhance the cellular uptake are polyamines and other polycationic moieties such as particular peptoids.

Recently, polycationic polyamines have been shown to be efficacious in the cellular delivery of oligonucleotides such as DNA [5–7] and RNA [8–11]. Conjugates of polyamines with aliphatic lipids or cholesterol yielding, i.e., dioctadecylaminoglycylspermine (DOGS, transfectam) are well established reagents for the transfection of DNA and oligonucleotides [5–7,11–13] displaying only very little toxicity towards mammalian cells [11]. They have also been shown to function in the recognition of biomacromolecules. Likewise, other polycationic species, such as the *N-*alkylated glycine oligomers (peptoids) [14–25], have emerged as powerful tools in the context of drug delivery [26–29], peptidomimetics and other biologically relevant applications [28,30–32] as well as materials science [33–34].

During the last decade the synthesis of polyamines and peptoids has been well established on solid phases [26–27,29,35–39]. However, the on-bead addition of oligosaccharide or monosaccharide modifications are not known so far. The modification of polyamines or peptoids is usually achieved by alternation of the termini [36,40] or by direct use of different side-chain functionalities. For peptoids, CuAAC has already been used successfully to introduce diverse side-chain functionalities directly during solid-phase synthesis of peptoids starting from both, azido- and alkyne-functionalized side chains [41–42]. In addition CuAAC has also been used in order to constrain peptoid secondary structures [43].

CuAAC reactions for the attachment of sugar residues to peptoid backbones have been reported for some cases [44–45]; however, a fully glycosylated structure is unknown (for glycodendrons see [46]). In this study, we describe the first solid-phase synthesis of glycosylated polyamines and a fully glycosylated hexapeptoid.

## Results and Discussion

### Synthesis of azidosugars

**Figure 1:**
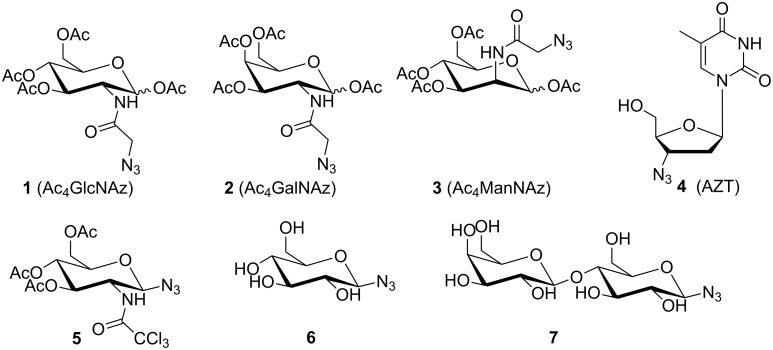
Azidosugars used in this study. The synthesis of the azidosugars **1**–**3** was modified from [47–48], compounds **4**, **6** and **7** were commercially available.

Although the syntheses of the azidosugars **1**–**3** and **5** (Figure 1) were described before [47], we optimized and revised the procedure reported by Laughlin and Bertozzi [48] due to some difficulties in obtaining reproducible results.

Instead of 5.00 equiv of chloroacetic anhydride only 1.10 equiv were used. A change from LiN_3_ to NaN_3_, which is more stable and cheaper, in conjunction with lowering the amount of the azide salt from 5.00 equiv to less than 3.60 equiv gave similar results. The solvent was changed from DMF to MeOH. By applying revised conditions 55% yield (on a 4.64 mmol scale) was obtained with a comparable yield on a 1.16 mmol scale (61%). In contrast to the originally reported procedure, the purification and isolation of intermediates could be omitted. We tried to use fewer equivalents of sodium azide during the scale-up of the reaction but this caused a decrease of the overall reaction yields.

In all cases, the test reactions were performed starting from D-glucosamine hydrochloride as a model compound for the synthesis of 1,3,4,6-tetra-*O*-acetyl-*N-*azidoacetyl-D*-*glucosamine (Ac_4_GlcNAz = **1**). Optimal variations from Bertozzi’s protocol for this model compound were the use of 1.10 equiv chloroacetic anhydride in the first step and 3.50 equiv sodium azide as well as methanol in the second step (for further details, see Table 1). Eventually, we applied these optimized reaction conditions to synthesize also 1,3,4,6-tetra-*O*-acetyl-*N-*azidoacetyl-D*-*galactosamine (Ac_4_GalNAz = **2**) and 1,3,4,6-tetra-*O*-acetyl-*N-*azidoacetyl-D*-*mannosamine (Ac_4_ManNAz = **3**) with similar yields under these optimized conditions (see Supporting Information File 1).

**Table 1:** Optimization of the synthesis of azide **1**.

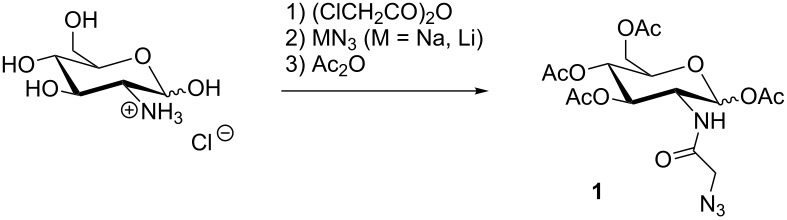				

Entry	Reaction scale	Solvent (2nd step)	MN_3_	Overall reaction yield (over 3 steps)

**1**	1.16 mmol	DMF	5.00 equiv (M = Li)	61%
**2**	4.64 mmol	DMF	5.00 equiv (M = Na)	23%
**3**	4.64 mmol	MeOH	2.10 equiv (M = Na)	35%
**4**	4.64 mmol	MeOH	3.50 equiv (M = Na)	55%
**5**	13.4 mmol	MeOH	2.10 equiv (M = Na)	38%
**6**	23.2 mmol	MeOH	2.00 equiv (M = Na)	42%

### Click reaction on alkynylated polyamines and peptoids

**Scheme 1:**
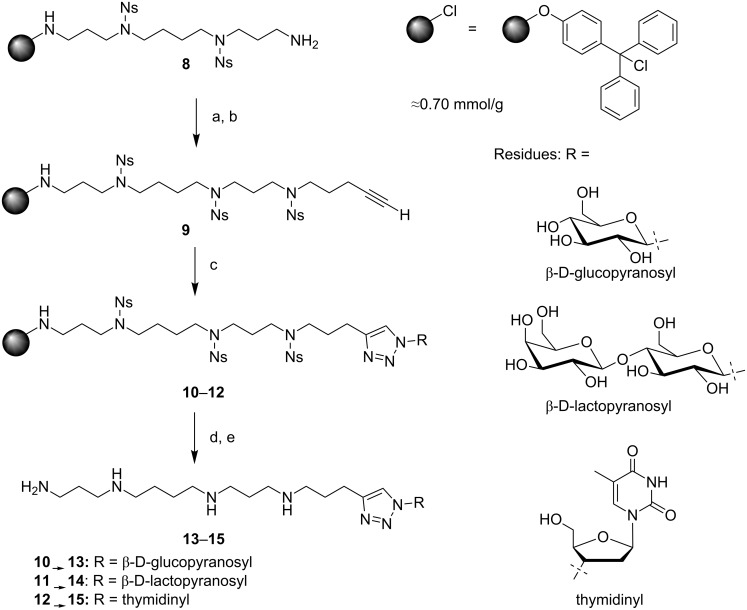
Reaction conditions and reagents: (a) Ns-chloride (6.00 equiv), 2,4,6-collidine (12.0 equiv), CH_2_Cl_2_, rt, 16 h; (b) 5-chloropent-1-yne (10.0 equiv), K_2_CO_3_ (15.0 equiv), DMF, 60 °C, 16 h; (c) azidosugars **6, 7** or AZT (**4**) (2.00 equiv/1.86 equiv), CuSO_4_·5H_2_O (0.500 equiv), sodium ascorbate (5.00 equiv), DMF/H_2_O (6:1), rt, 2 d; (d) DBU (20.0 equiv), β-mercaptoethanol (20.0 equiv), DMF, rt, 18 h; (e) 1% TFA in CH_2_Cl_2_, rt, 10 min.

The covalent coupling of many biomacromolecules to solid-phase-bound polycationic moieties, such as polyamines or peptoids, often requires a hydrophilic reaction environment as well as very mild cleaving conditions of the final product from the solid support. To avoid a degradation of the coupled biomacromolecules at high concentrations of strong acids, a polystyrene resin was chosen that contains a tritylchloride linker. The resin was obtained by treatment of Merrifield resin with *p*-hydroxytriphenylmethyl alcohol and subsequent chlorination [49–50]. This tritylchloride linker allowed a mild cleavage of the acid labile products using less than 0.5% trifluoroacetic acid (TFA) in dichloromethane. The loading of the Merrifield resin occurred in pretty good yields as the ratio of the measured to the calculated loading value was 0.70 mmol/g to 0.78 mmol/g (Scheme 1).

The synthesis started with the assembly of the 2-nitrobenzenesulfonyl-(Nosyl, further abbreviated with Ns)-protected spermine backbone **8** on a solid phase via Fukuyama Ns strategy [51]. The next step was the Ns protection of the residual primary amine with 6.00 equiv of Ns-chloride and 12.0 equiv 2,4,6-collidine in CH_2_Cl_2_ followed by *N*-alkylation with 5-chloropent-1-yne to insert the terminal alkyne moiety. To accomplish that, we used 10.0 equiv of alkyne and 15.0 equiv of K_2_CO_3_ in DMF; the reaction led to resin **9** with virtually quantitative yield, as shown in Scheme 1. For the CuAAC with the azides moieties **4**, **6** and **7**, respectively, we used 0.500 equiv of CuSO_4_·5H_2_O and 5.00 equiv sodium ascorbate as the catalytic system, which is a slightly higher catalyst concentration than reported for the reaction in solution [52]. To ensure, that the reaction proceeds completely we chose 2 days of agitation at ambient temperature and obtained resin **10**−**12**.

Finally, the Ns deprotection was achieved in 18 h with 20.0 equiv of 1,8-diazabicyclo[5.4.0]undec-7-ene (DBU) and 20.0 equiv of β-mercaptoethanol. The cleavage from the resin was carried out with 1% TFA in dichloromethane at 10 min residence time. The products **13**–**15** were obtained with >90% yield, calculated on the initial loading of the resin (0.70 mmol/g). The ^1^H NMR data clearly showed the aromatic shift of the triazole proton at 8.02 ppm, 8.03 ppm and 7.94 ppm for **13**–**15**, respectively. The ^13^C NMR spectra indicated the presence of the anomeric carbon atoms at 89.70 ppm for **13** as well as at 89.41 ppm and 105.47 ppm for **14**.

**Scheme 2:**
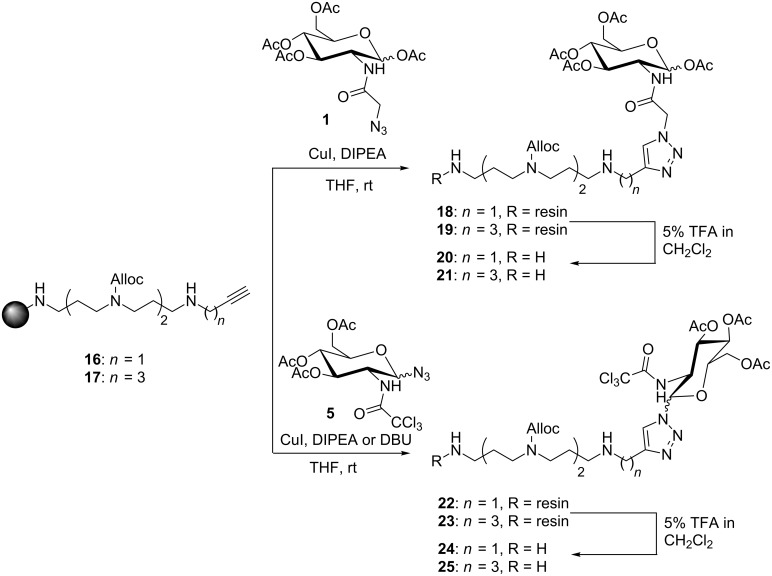
Synthesis of spermine conjugates **20**,**21** and **24**,**25**. 2-chlorotrityl chloride resin was used as a solid support.

In a different approach, we synthesized glycosylated spermine derivatives by our optimized procedure on a 2-chlorotrityl chloride resin [35,37–39]. The reaction of the resin **16** and **17** with the Ac_4_GlcNAz derivative **1** proceeded smoothly in the presence of copper ions. In some cases, changing the base from DIPEA to DBU was beneficial (Scheme 2).

After these encouraging results, we turned our attention to peptoids. For the glycosylation of peptoids, we envisaged the generation of a fully glycosylated peptoid in order to investigate the compatibility of peptoid synthesis and decoration with sugars.

**Scheme 3:**
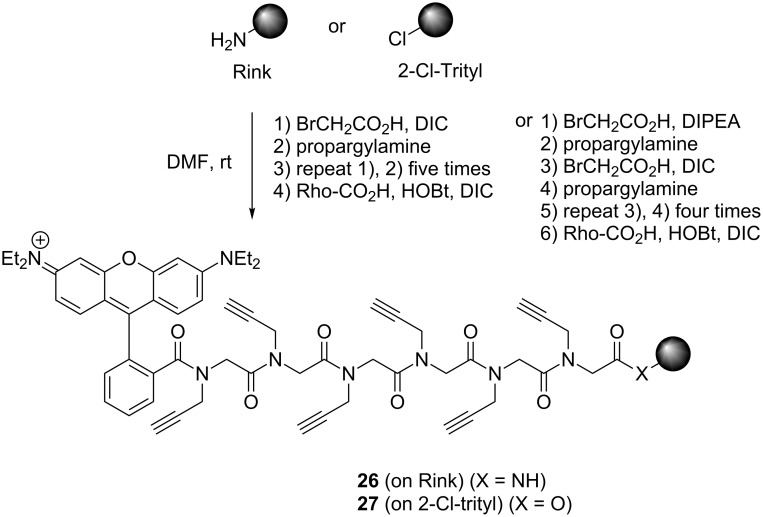
Synthesis of hexaalkynyl peptoids **26** and **27** on solid supports.

As a model compound we started with the synthesis of a hexaglycosylated peptoid hexamer. Therefore, we synthesized a hexaalkynated peptoid structure **26**,**27** on resin (Scheme 3).

The synthesis of a hexaalkynated peptoid backbone **26**,**27** was carried out on Rink or Barlos resin containing a 2-chlorotrityl chloride linker by standard methods using the submonomer strategy [14]. By using this method, the peptoid backbone is assembled in two subsequently repeated steps: In the first step (acylation), bromoacetic acid is reacted with the resin, and in the second step (amination) a primary amine is used to substitute the bromine to give the peptoid residue. This approach avoids the use of *N-*terminally protected monomers, which have to be synthesized in advance.

For the incorporation of the alkyne side chains we chose propargylamine as building block. A sixfold repetitive coupling sequence resulted in the peptoids, which were further modified with rhodamine B (Rho-CO_2_H) as an easily accessible and versatile fluorescent tag.

Rhodamine B was coupled to the *N*-terminus in order to provide a label for future biological applications, such as the study of the cellular uptake. For both resins, the peptoid synthesis was successful; the only differences are the functional groups on the *C*-terminus. After cleavage from Rink-amide resin with trifluoroacetic acid, an amide is obtained, whereas cleavage from 2-chlorotrityl chloride resin with hexafluoroisopropanol (HFIP) gives the carboxylic acid. The mild cleavage conditions of the 2-chlorotrityl linker did not harm the sugar moieties of the final glycosylated compound, and this linker was therefore favored over the Rink linker.

**Scheme 4:**
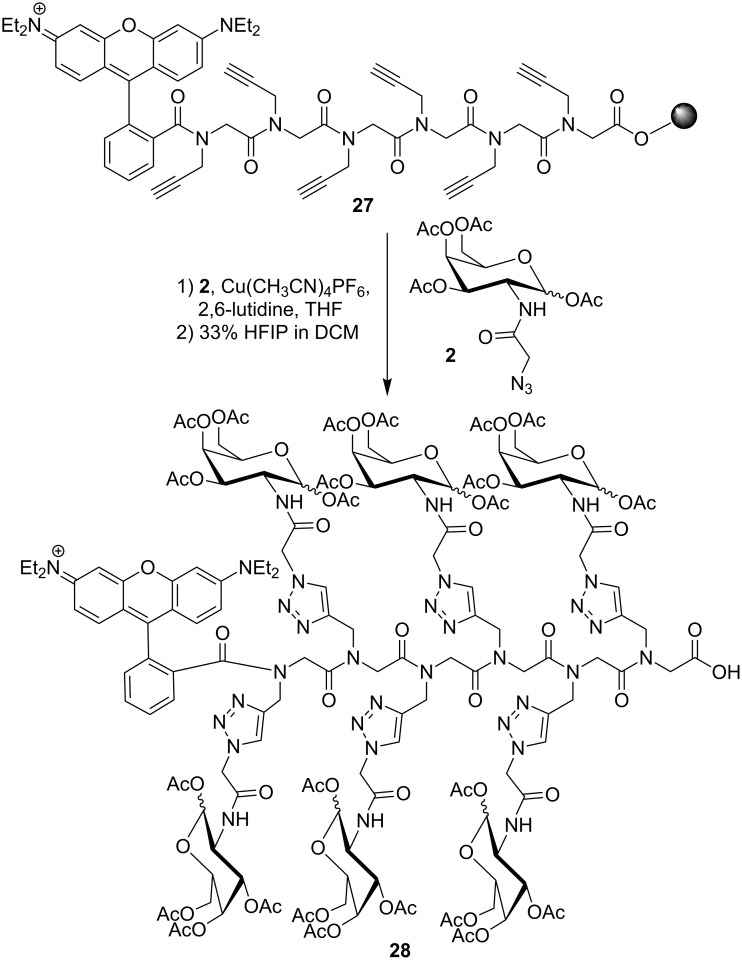
Synthesis of a hexa-glycosylated peptoid **28**.

For the conjugation of Ac_4_GalNAz (**2**), the same conditions were used as described for the spermine conjugation (Scheme 2) with minor modifications. The CuAAC was carried out by using Cu(CH_3_CN)_4_PF_6_ in THF with 2,6-lutidine as base. Only 1.60 equiv of the azidosugar were necessary to achieve full conversion after 18 h, and no shorter oligomers were observed in the MALDI–TOF spectrum. Cleavage from the resin by treatment with 33% hexafluoroisopropanol (HFIP) in dichloromethane and subsequent HPLC purification resulted in the fully glycosylated hexameric product **28** (Scheme 4).

## Conclusion

In conclusion, we were able to improve protocols for the synthesis of tetra-*O*-acetyl protected sugars. Applying this, better yields as well as upscaling was possible. With these sugar building blocks, functionalization of polyamine derivatives was possible directly on solid supports by using copper-catalyzed alkyne azide cycloaddition conditions. In addition to that, the functionalization of a peptoid-hexaalkyne was also possible. By using the more labile 2-chlorotrityl chloride resin, the cleavage of peptoid **28** could be achieved without degradation.

## Supporting Information

File 1 Methods and NMR spectra.
