# Supplementary material for: Peptoids and polyamines going sweet: Modular synthesis of glycosylated peptoids and polyamines using click chemistry
Source: Beilstein J Org Chem. 2013 Jan 10;9:56–63. doi: 10.3762/bjoc.9.7 (PMC3566861; doi:10.3762/bjoc.9.7)
Supplement: File 1 — Methods and NMR spectra. [file Beilstein_J_Org_Chem-09-56-s001.pdf]

Supporting Information

for

Peptoids and polyamines going sweet: Modular synthesis of glycosylated peptoids and polyamines using click chemistry

Daniel Fürniss[1], Timo Mack[2], Frank Hahn[2], Sidonie B. L. Vollrath[1], Katarzyna Koroniak[1], Ute Schepers*[3] and Stefan Bräse*[1,3]

Address: [1]Institute of Organic Chemistry, Karlsruhe Institute of Technology (KIT), Fritz-Haber-Weg 6, 76131 Karlsruhe, Germany, [2] Kekulé Institute of Organic Chemistry and Biochemistry, Rheinische Friedrich Wilhelms University of Bonn, Germany and [3]Institute of Toxicology and Genetics, Karlsruhe Institute of Technology (KIT), Hermann-von-Helmholtz-Platz 1, 76344 Eggenstein-Leopoldshafen, Germany.

Email: Stefan Bräse – braese@kit.edu; Ute Schepers – ute.schepers@kit.edu

* Corresponding author

Methods and NMR spectra

General methods

All chemicals were obtained from commercial suppliers (Merck, Sigma-Aldrich, Fluka, Acros, ABCR, Alfa Aesar, KMF), the azidosugars, 1-azido-1-deoxy-β-D-glucopyranoside and 1-azido-1-deoxy-β-D-lactopyranoside as well as AZT were purchased from Sigma Aldrich. The solvents were anhydrous and of reagent grade. Merrifield resin was purchased from Polymer Laboratories (PL-CMS resin,

0.97 mmol/g, 1% crosslink, 75–150 µm mesh). 2-Chlorotrityl chloride resin was purchased from Agilent (PL-Cl-Trt-Cl resin, 2.06 mmol/g, 1% crosslink, 150–300 µm mesh). Rink amide resin was purchased from Novabiochem (0.64 mmol/g, 1% crosslink, 100–200 µm mesh). All solid-phase reactions were typically carried out in glass frits under argon atmosphere and, if not mentioned otherwise, at ambient temperature. All solution-phase reactions were carried out in flasks with magnetic stirring. Reactions under inert gas were carried out in flasks equipped with septa under argon (supplied by using a standard manifold with vacuum and argon lines). NMR spectra were recorded at 25 °C by using Bruker AM 400 (400 (^{1}H) and 100 MHz (^{13}C)), Bruker DP300 (300 (^{1}H) and 75 MHz (^{13}C)), and DP400 (400 (^{1}H) and 100 MHz (^{13}C)) spectrometers. Due to rotamers, NMR spectra of peptoids were not recorded. All spectra are referenced to tetramethylsilane as the internal standard (δ = 0 ppm) by using the signals of the residual protons of $CHCl_3$ (7.26 ppm (^{1}H) or 77.0 ppm (^{13}C)) in $CDCl_3$, or CHD_2OD (3.31 ppm (^{1}H) or 49.1 ppm (^{13}C)) in CD_3OD. Multiplicities of signals are described as follows: s = singlet, bs = broad singlet, d = doublet, t = triplet, q = quartet, m = multiplet. Coupling constants (*J*) are given in hertz (Hz). Multiplicities in the ^{13}C NMR spectra were determined by DEPT (distortionless enhancement by polarization transfer) measurements. Mass spectra (ESI) were obtained by using an Agilent 6230 TOF LC/MS. QToF electrospray-ionisation (ESI) MS was performed with a Micromass QTof2 spectrometer. MALDI-TOF mass spectra were obtained by using either a Bruker Biflex IV spectrometer with a pulsed ultraviolet nitrogen laser, 200 µJ at 337 nm and a time-of-flight mass analyzer with a 125 cm linear flight path, or a Micromass TOFSpecE spectrometer in reflectron mode. 2,5-Dihydroxybenzoic acid and α-cyano-4-hydroxy cinnamic acid were used as the matrix. HPLC was performed on a Jasco HPLC system, with a C18 column (30 × 190 mm). Flow rate: 15 mL/min; solvent A: 0.1% TFA in water; solvent B: 0.1%

TFA in MeCN. Analytical TLC was performed on MERCK ready-to-use plates with silica gel 60 (F254). Column chromatography: MERCK silica gel 60, 0.04–0.063 mm. The analytical data of resin-bound substrates were taken from the unpurified free product after test cleavage from the resin.

General procedures for solid-phase synthesis

General washing procedure for resin. Procedure A: the resin was washed three times each with 10% H_2O in THF, THF/Et_2O and Et_2O. Procedure B: the resin was washed three times each with DMF, 0.1 M EDTA solution in H_2O, 10% H_2O in THF, THF/Et_2O and Et_2O. In the steps with two solvents they were used alternatingly. Per 1 g of the resin, 50 mL solvent was used.

Nosyl (Ns) deprotection. For cleavage of the Ns protecting group the resin was swelled in DMF for 15 min. Then, 20.0 equiv of β-mercaptoethanol and 20.0 equiv of DBU were added. After agitation overnight the reaction mixture was removed and the resin was washed according to procedure A.

Cleavage from the resin. The product was cleaved from the resin by adding 1% TFA in CH_2Cl_2. Meanwhile the color of the resin turned to red. After 5–10 min the product was washed off with CH_2Cl_2 and MeOH, followed by evaporation of the solvent in high vacuum.

Synthesis of azidosugars 1–3: To a solution of D-mannosamine/ D-glucosamine/ D-galactosamine hydrochloride (1.00 g; 4.64 mmol) in methanol (50 mL), sodium methanolate (30% w/w NaOMe in MeOH, 1.66 mL, 4.64 mmol, 1.00 equiv) was added and the mixture was stirred at room temperature for 30 min until complete dissolution. NEt_3 (0.47 g; 4.64 mmol, 1.00 equiv) and chloroacetic anhydride (871 mg; 5.10 mmol, 1.10 equiv) were added to the solution, and stirred at room

temperature overnight. The solvent was evaporated and the crude product was used in subsequent reaction without further purification. Comment: *If necessary, sodium bicarbonate was added to neutralize the solution.*

To a solution of *N*-chloroacetylmannosamine/ *N*-chloroacetylglucosamine/ *N*-chloroacetylgalactosamine in MeOH/H_2O (10:1, (20 mL /2 mL)) sodium azide NaN_3 (1.06 g; 16.24 mmol, 3.50 equiv) was added. The mixture was stirred for 5 h at 65 °C. Subsequently, the reaction mixture was concentrated and dried in vacuo. The residue was then suspended in pyridine (20 mL) and acetic anhydride (20 mL) was added to the solution. The reaction was stirred at room temperature overnight. After concentration in vacuo, the residue was dissolved in EtOAc (50 mL) and washed with 1N HCl, $NaHCO_3$ and brine (each 50 mL) (*CAUTION*: *extraction with sodium bicarbonate causes gas formation and excess pressure in the separating funnel*). After drying over $MgSO_4$ the crude product was purified by column chromatography (cyclohexane/ethyl acetate 1:1) to afford the desired product (mixture of α/β anomers) as a white-yellowish oil that solidified upon *lyophilization.* The yield for all azides is about 55% overall yield.

1,3,4,6-Tetra-*O*-acetyl-*N*-azidoacetyl-D-glucosamine (1); mixture of anomers: α/β : 65/35; ^{1}H NMR (400 MHz, $CDCl_3$): δ = 6.49 (d, 1H, *J* = 9.3 Hz, NH-β), 6.43 (d, 1H, *J* = 9.3 Hz, NH-α), 6.19 (d, 1H, *J* = 3.7 Hz, H-1α) 5.80 (d, 1H, *J* = 8.7 Hz, H-1β), 5.32–5.18 (m, 3H), 5.13 (t, 1H, *J* = 9.6 Hz, *J* = 9.6 Hz), 4.44 (ddd, 1H, *J* = 3.7 Hz, *J* = 8.9 Hz, *J* = 10.8 Hz), 4.29–4.17 (m, 3H), 4.14 (d, 1H, *J* = 2.2 Hz), 4.10 (d, 1H, *J* = 2.2 Hz), 3.93 (s, 2H), 3.91 (s, 2H), 3.83 (ddd, 1H, *J* = 2.3 Hz, *J* = 4.6 Hz, *J* = 9.8 Hz), 2.20 (s, 3H), 2.11 (s, 3H), 2.09 (s, 3H), 2.05 (s, 3H), 2.04 (s, 3H), 2.04 (s, 3H). ^{13}C NMR (100.6 MHz, $CDCl_3$): δ = 171.5, 170.8, 170.6, 170.6, 169.3, 169.2, 169.1, 168.7, 168.0, 166.8, 92.2 (C-1β), 90.2 (C-1α), 72.9, 72.1, 70.3, 69.8, 67.7, 67.4, 61.6,

61.5, 53.2, 52.6, 52.4, 51,2, 20.9, 20.8, 20.7, 20.6, 20.6, 20.5; MS (ESI), $[M + Na]^+$ $C_{16}H_{22}N_4NaO_{10}$: calcd. 453.1234; found 453.1471.

1,3,4,6-Tetra-*O*-acetyl-*N*-azidoacetyl-D-galactosamine (2); mixture of anomers: α/β : 65/35; ^{1}H NMR (400 MHz, $CDCl_3$): δ = 6.39 (d, 1H, *J* = 9.4 Hz, NH-β), 6.29 (d, 1H, *J* = 9.1 Hz, NH-α), 6.23 (d, 1H, *J* = 3.5 Hz, H-1α), 5.79 (d, 1H, *J* = 8.8 Hz, H-1β), 5.45 (d, 1H, *J* = 2.0 Hz), 5.39 (d, 1H, *J* = 3.2 Hz), 5.26 (ddd, 1H, *J* = 3.2 Hz, *J* = 11.5 Hz, *J* = 23.1 Hz), 4.74–4.69 (m, 1H), 4.67 (d, 1H, *J* = 3.6 Hz), 4.38 (td, 1H, *J* = 9.2 Hz, *J* = 11.2 Hz), 3.95 (s, 2H), 3.92 (s, 2H), 2.18 (s, 3H), 2.16 (s, 3H), 2.12 (s, 3H), 2.05 (s, 3H), 2.04 (s, 3H), 2.03 (s, 3H); ^{13}C NMR (100.6 MHz, $CDCl_3$): δ = 170.9, 170.8, 170.4, 170.4, 170.4, 170.3, 170.1, 169.9, 169.3, 169.0, 168.8, 167.2, 166.9, 166.9, 93.8, 92.6, 90.9, 73.9, 71.8, 70.2, 69.9, 68.7, 67.6, 66.6, 66.6, 62.1, 61.2, 56.2, 52.6, 52.5, 49.9, 47.0, 21.0, 20.9, 20.8, 20.8, 20.7, 20.6, 20.6; MS (ESI), $[M + Na]^+$ $C_{16}H_{22}N_4NaO_{10}$: calcd. 453.1234; found. 453.1451.

1,3,4,6-Tetra-*O*-acetyl-*N*-azidoacetyl-D-mannosamine (3); mixture of anomers: α/β : 54/46; ^{1}H NMR (400 MHz, $CDCl_3$): δ (ppm) = 6.67 (d, 1H, *J* = 9.0 Hz); 6.63 (d, 1H, *J* = 9.3 Hz); 6.03 (d, 1H, *J* = 1.8 Hz); 5.88 (d, 1H, *J* = 1.6 Hz); 5.33 (dd, 1H, *J* = 10.2 Hz, *J* = 4.3 Hz); 5.21 (t, 1H, *J* = 10.0 Hz); 5.15 (t, 1H, *J* = 9.8 Hz); 5.05 (dd, 1H, *J* = 9.9 Hz, *J* = 3.9 Hz); 4.72 (ddd, 1H, *J* = 9.0 Hz, *J* = 3.8 Hz, *J* = 1.6 Hz); 4.61 (ddd, 1H, *J* = 9.3 Hz, *J* = 4.2 Hz, *J* = 1.9 Hz); 4.27–4.19 (m, 2H); 4.16–3.99 (m, 7H); 3.81 (ddd, 1H, *J* = 9.6 Hz, *J* = 4.6 Hz, *J* = 2.5 Hz); 2.17 (s, 3H); 2.10 (s, 3H); 2.10 (s, 6H); 2.05 (s, 6H); 1.99 (s, 3H); 1.99 (s, 3H); ^{13}C NMR (100 MHz, $CDCl_3$): δ (ppm) = 170.5; 170.1; 170.1; 169.6; 168.3; 168.1; 167.4; 166.8; 91.3; 90.3; 73.4; 71.4; 70.3; 68.8; 65.1; 65.0; 61.8; 61.7; 52.6; 52.4; 49.7; 49.3; 20.8; 20.7; 20.7; 20.6; 20.6; MS (ESI), $[M + Na]^+$ $C_{16}H_{22}N_4NaO_{10}$: calcd. 453.1234; found 453.1481.

Sperminyl-*N*-[(*N*',*N*'',*N*'''-trinosyl)-*N*'''-pent-4-ynyl]*p*-hydroxytrityl resin 9. To a suspension of 2.43 g (1.00 equiv) resin **8** and 2.09 mL (12.0 equiv) of 2,4,6-collidine in 15 mL CH_2Cl_2 was added 1.80 g (6.00 equiv) of 2-nitrobenzenesulfonylchloride. The resin was agitated for 16 h, then washed according to procedure A and dried under reduced pressure. After drying, 1.94 g (1.00 equiv) resin and 1.45 g (15.0 equiv) K_2CO_3 were suspended together in 20 mL DMF. Then, 1.0 mL (10.0 equiv) of 5-chloropent-1-yne was added and the mixture was agitated at 60 °C for 16 h. After cooling down, the solvent was removed and the resin was washed following procedure A until all K_2CO_3 was removed. An orange resin was obtained after drying under reduced pressure. NMR data and MS were obtained after cleavage from the resin according to the general procedures for solid-phase synthesis.

^{1}H NMR (400 MHz, [D_4]-MeOH): δ (ppm) = 1.53 (m, 4 H, CH_2); 1.71 (m, 3H, CH_2); 1.96 (m, 4H, CH_2); 2.15 (m, 2H, CH_2); 3.05 (m, 4H, CH_2); 3.32–3.49 (m, 10H, CH_2); 7.72–7.89 (m, 9H, CH_{ar}); 7.97–8.10 (m, 3H, CH_{ar}); MS (MALDI) *m*/*z*: 824.15 $[M + H]^+$.

Sperminyl-*N*-(*N*'''-1-β-D-glucopyranosyl-4-propyl-1*H*-1,2,3-triazole) 13. A suspension of 200 mg (1.00 equiv) resin **9** in 2 mL DMF reacted with 12.3 mg (0.500 equiv) $CuSO_4{\cdot}5H_2O$ in 1 mL DMF, 97.72 mg (5.00 equiv) sodium ascorbate in 0.5 mL aqua dest. and 40.08 mg (2.00 equiv) 1-azido-1-deoxy-β-D-glucopyranosid or 71.75 mg (2.00 equiv). After agitating for 2 d the reaction mixture was removed and the resin was washed according to procedure B. After deprotection of the nosyl group and washing of the resin according to procedure A the products were cleaved from the resin according to general procedures.

^{1}H NMR (400 MHz, [D4]-MeOH): δ (ppm) = 1.71–1.81 (m, 6H, C*H2*); 2.01–2.15 (m, 6H, C*H2*); 3.05–3.15 (m, 14H, C*H2*); 3.35 (m, 2H, C*H*); 3.45–3.60 (m, 1H, C*H*); 3.72 (dd, 1H, C*H2*, J_1 = 12.15 Hz, J_2 = 5.22 Hz,); 3.88 (m, 1H, C*H2*); 5.58 (d, 1H, C*H*, *J* =

9.19 Hz); 8.02 (s, 1H, C*H*$_{ar}$, (Triazole-H)); ^{13}C NMR (100 MHz, [D4]-MeOH): δ (ppm) = 24.17; 24.90; 25.33; 27.46; 29.94; 33.72; 37.80; 39.33; 45.83; 62.26; 70.84; 73.96; 78.35; 81.00; 89.70, 122.78, 147.36; MS (ESI) *m*/*z*: 474.34 [M + H]$^+$; HRMS (EI, $C_{21}H_{44}O_5N_7$): calc. 474.3404; found 474.3401.

Sperminyl-*N*-(*N*'''-1-β-D-lactopyranosyl-4-propyl-1*H*-1,2,3-triazole) 14. A suspension of 200 mg (1.00 equiv) resin **9** in 2 mL DMF reacted with 12.3 mg (0.500 equiv) $CuSO_4·5H_2O$ in 1 mL DMF, 97.72 mg (5.00 equiv) sodium ascorbate in 0.5 mL aqua dest. and 71.75 mg (2.00 equiv) 1-azido-1-deoxy-β-D-lactopyranoside. After agitating for 2 d the reaction mixture was removed and the resin was washed according to procedure B. After deprotection of the nosyl group and washing of the resin according to procedure A the products were cleaved from the resin according to general procedures.

^{1}H NMR (400 MHz, [D$_4$]-MeOH): δ (ppm) = 1.69–1.81 (m, 6H, C*H$_2$*); 2.01–2.15 (m, 6H, C*H$_2$*); 3.04–3.16 (m, 14H, C*H$_2$*); 3.33–3.36 (m, 2H, C*H*); 3.50–3.65 (m, 5H, C*H$_2$*+C*H*); 3.72–3.90 (m, 5H, C*H$_2$*+C*H*); 4.00 (m, 1H, C*H*); 4.43 (d, 1H, C*H*, *J* = 7.55 Hz); 5.63 (d, 1H, C*H*, *J* = 9.32 Hz); 8.03 (s, 1H, C*H*$_{ar}$, (triazole-H)); ^{13}C NMR (100 MHz, [D$_4$]-MeOH): δ (ppm) = 24.18; 24.91; 25.33; 27.46; 29.95; 33.72; 37.81; 39.34; 45.83; 61.65; 62.71; 70.47; 72.68; 73.85; 74.94; 76.96; 77.30; 79.66; 79.94; 89.41, 105.27; 123.04; 147.26; MS (ESI) *m*/*z*: 636.40 [M + H]$^+$; HRMS (EI, $C_{27}H_{54}O_{10}N_7$): calc. 636.3932; found 636.3935.

Sperminyl-*N*-(*N*'''-1-2',3'-deoxythymidine-4-propyl-1*H*-1,2,3-triazole) 15. 402.5 mg (1.00 equiv) of resin **9** was suspended in 2 mL DMF. Then, 25.2 mg (0.500 equiv) $CuSO_4·5H_2O$ in 1 mL DMF and 201.9 mg (5.00 equiv) sodium ascorbate in 0.5 mL distilled water were added, followed by 100 mg (1.86 equiv) AZT.

The suspension was agitated for 2.5 d, then the reaction mixture was removed from the resin. After that, the orange resin was washed according to procedure B, deprotected from the Nosyl group and dried under vacuum.

^{1}H NMR (400 MHz, [D_4]-MeOH): δ (ppm) = 1.71 (m, 2H, CH_2); 1.81 (m, 4 H, CH_2); 1.90 (d, 3 H, CH_3, J = 1.14 Hz); 2.10 (m, 6H, CH_2); 2.13 (m, 2H, CH_2); 2.85 (m, 2H, CH_2); 3.03–3.15 (m, 12H, CH_2); 3.77 (dd, 1H, CH_2, J_1 = 12.18 Hz, J_2 = 3.22 Hz); 3.89 (dd, 1H, CH_2, J_1 = 12.13 Hz, J_2 = 3.03 Hz); 4.35 (m, 1H, CH); 5.39 (dt, 1H, CH, J_1 = 8.67 Hz, J_2 = 4.93 Hz); 6.49 (t, 1H, CH, J = 6.57 Hz); 7.90 (d, 1H, CH_{ar}, J = 1.01 Hz); 7.94 (s, 1H, CH_{ar}, (triazole-H)); MS (ESI) *m*/*z*: 536.36 $[M + H]^+$.

Synthesis of 20

In a vial 0.180 mmol of alkyne resin **16** was swollen in 6 mL abs. THF. 0.360 mmol of azidosugar **1**, 0.360 mmol of copper(I) iodide and 9.00 mmol DIPEA were added. The vial was sealed and shaken for 16 h at room temperature. Afterwards, the solvent was removed and the resin was washed with THF, saturated aqueous sodium ascorbate solution, water, THF/MeOH/THF/MeOH/THF, MeOH/CH_2Cl_2/MeOH/CH_2Cl_2/MeOH, and three times with CH_2Cl_2. The resin was dried in vacuo. After cleavage from the resin with 5% TFA in dichloromethane, the product was obtained.

MS (MALDI-TOF): *m*/*z* = 838.9 $[M + H]^+$.

Synthesis of hexaalkyne 27 on 2-chloro tritylchloride resin

For the synthesis, 50 mg (0.103 mmol, 1.00 equiv) of 2-chlorotrityl chloride resin and washed in a fritted plastic syringe (Multisyntech) with 1 mL of dichloromethane, followed by swelling in 1 mL of dichloromethane for 5 min. The first submonomer was added by reacting 77.2 mg of bromoacetic acid (0.555 mmol, 5.40 equiv) and 99 µL of DIPEA (71.8 mg, 0.555 mmol, 5.00 equiv) in 1 mL of dichloromethane on a shaker

platform for 40 min at room temperature, followed by washing with dichloromethane (three times with 2 mL) and DMF (three times with 2 mL). The bromoacetylated resin was incubated with 1 mL of 47.0 mg propargylamine (0.855 mmol, 8.30 equiv) in 1 mL DMF on a shaker platform at room temperature, followed by washing with DMF (three times with 3 mL). The displacement reaction times were 30 min for propargylamine. The following bromoacetylations were carried out by reacting the resin with 143 mg bromoacetic acid (1.03 mmol, 10.0 equiv) and 160 µL DIC (130 mg, 1.03 mmol, 10.0 equiv) in 1 mL DMF for 30 min. Coupling steps were continued until the desired peptoid length was achieved. After the last displacement step, the resin was washed with DMF/dichloromethane/DMF (two times with 3 mL for each solvent).

For the coupling of rhodamine B, the resin was incubated for 18 h with a solution of 144 mg rhodamine B (0.309 mmol, 3.00 equiv), 40.5 mg HOBt (0.309 mmol, 3.00 equiv) and 76.4 µL DIC (61.9 mg, 0.309 mmol, 3.00 equiv) in 2 mL DMF on a shaker platform. After the reaction the resin was washed with DMF and dichloromethane until the washing solution was colorless.

Mass spectrum (MALDI): calc.: 1013.46 g/mol, found: 1013.3 $[M]^+$.

Cu-catalyzed alkyne azide cycloaddition on resin 27 and cleavage from the resin

For the reaction with the sugar, the resin **27** was swollen in THF, and 70 mg $Cu(CH_3CN)_4PF_6$ (0.188 mmol, 1.83 equiv) was directly weighed into the plastic syringe containing the resin. In 2 mL dry THF, 310 mg of Ac_4GalNAz (**2**, 0.721 mmol, 7.00 equiv) and 100 µL 2,6-lutidine (92.0 mg, 0.859 mmol, 8.34 equiv) were dissolved and added to the resin. The resin was shaken for 18 h at rt.

After the reaction, the resin was washed with saturated aqueous sodium ascorbate solution, water, THF and dichloromethane (three times 2 mL each).

The peptoid was cleaved from the resin using 3 mL 33% HFIP in dichloromethane (*v*/*v*) at room temperature for 30 min. After collecting the cleavage solution, the resin was washed with 3 mL dichloromethane twice. The elution and washing solutions were combined. The solvent was evaporated under a stream of argon gas. After cleavage, HPLC purification and lyophilization, 70 mg (0.019 mmol, 19% yield) of a purple solid (**28**) were obtained.

Mass spectrum (MALDI, *Matrix*: DHB): calc.: 3594 g/mol, found: 3593 $[M]^+$; Analytical HPLC (5−95% Acetonitrile +0.1% TFA in 30 min, t_{Ret} = 15.9 min, Detection at 218 nm), Purity: 92%.

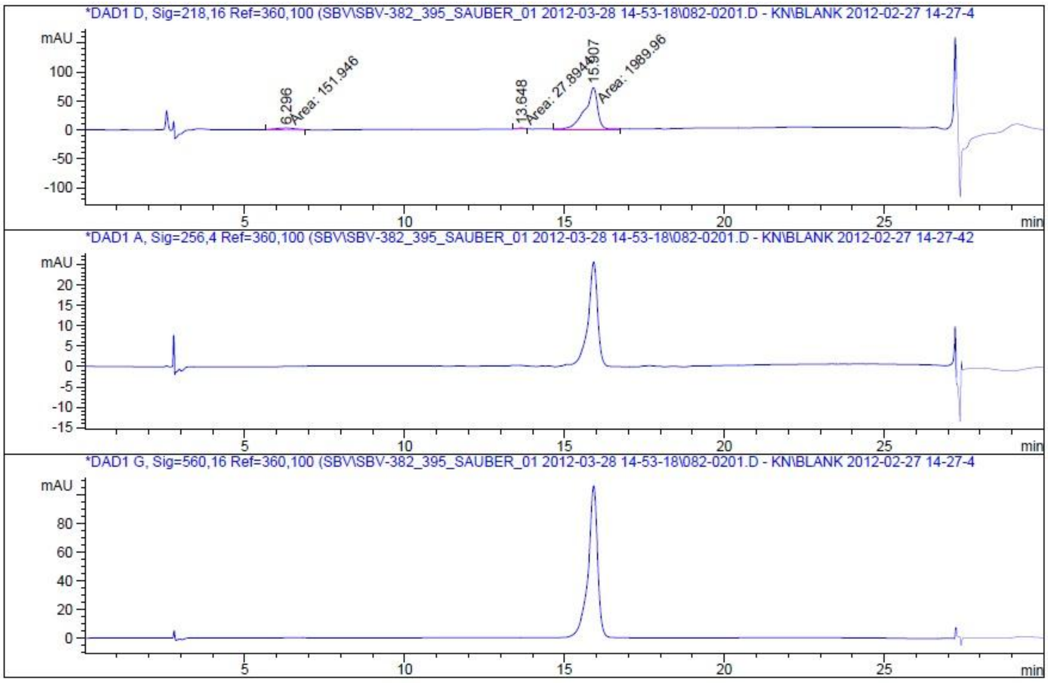

NMR spectra:

^{1}H NMR of compound 9 (after cleavage from resin):

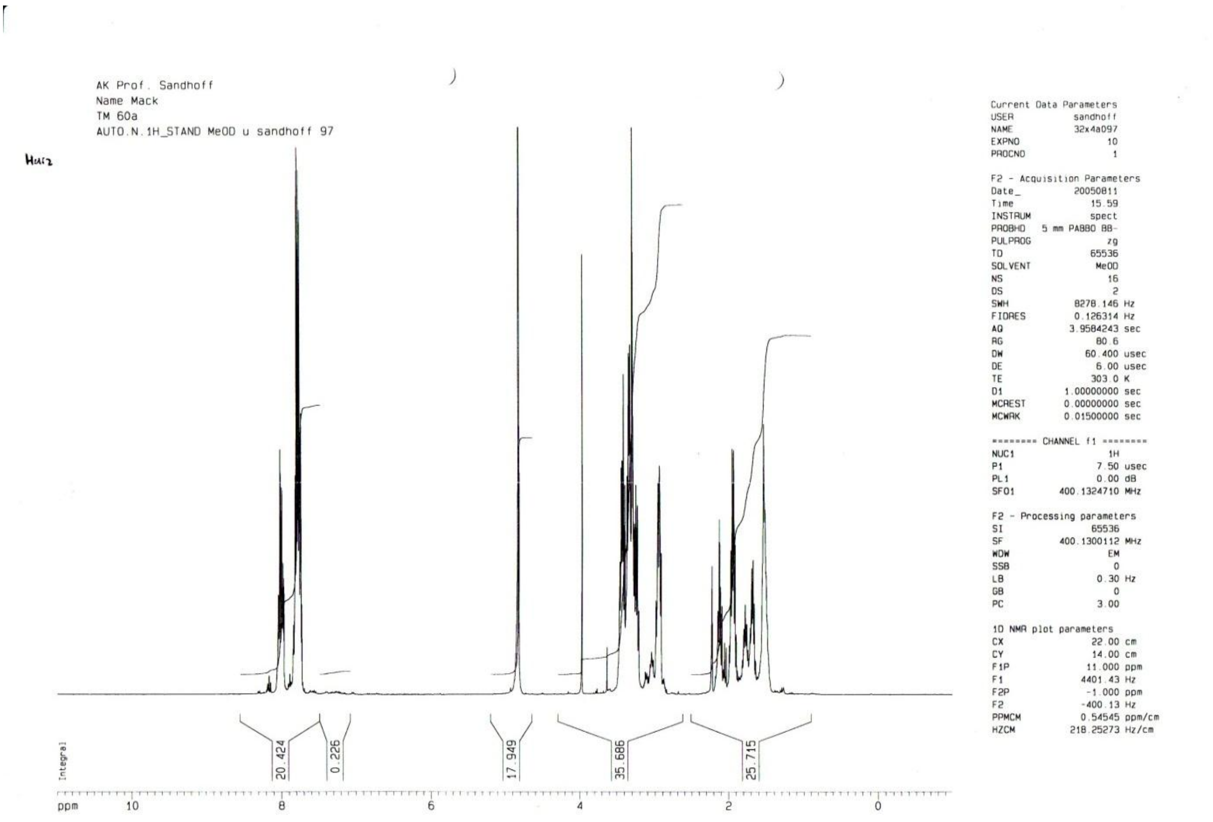

^{13}C NMR of compound 9 (after cleavage from resin)

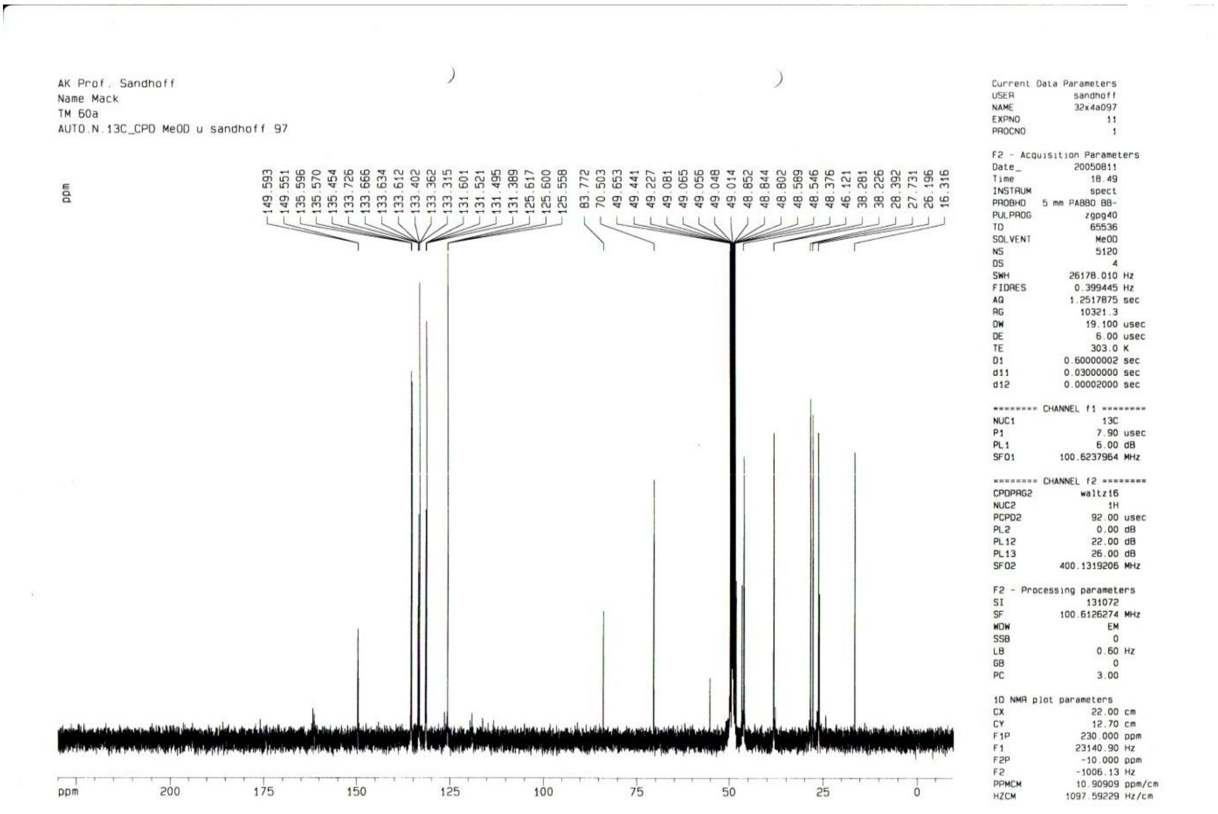

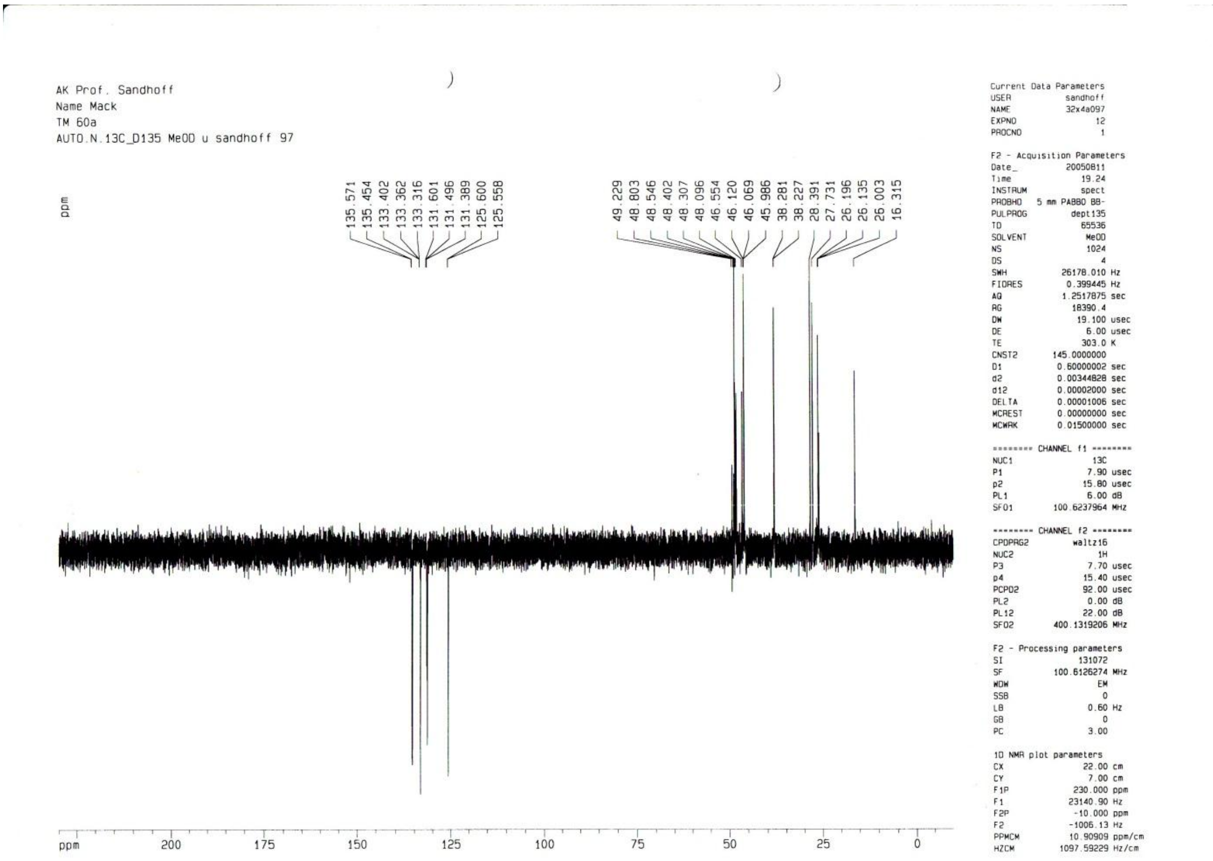

^{1}H NMR of compound 13:

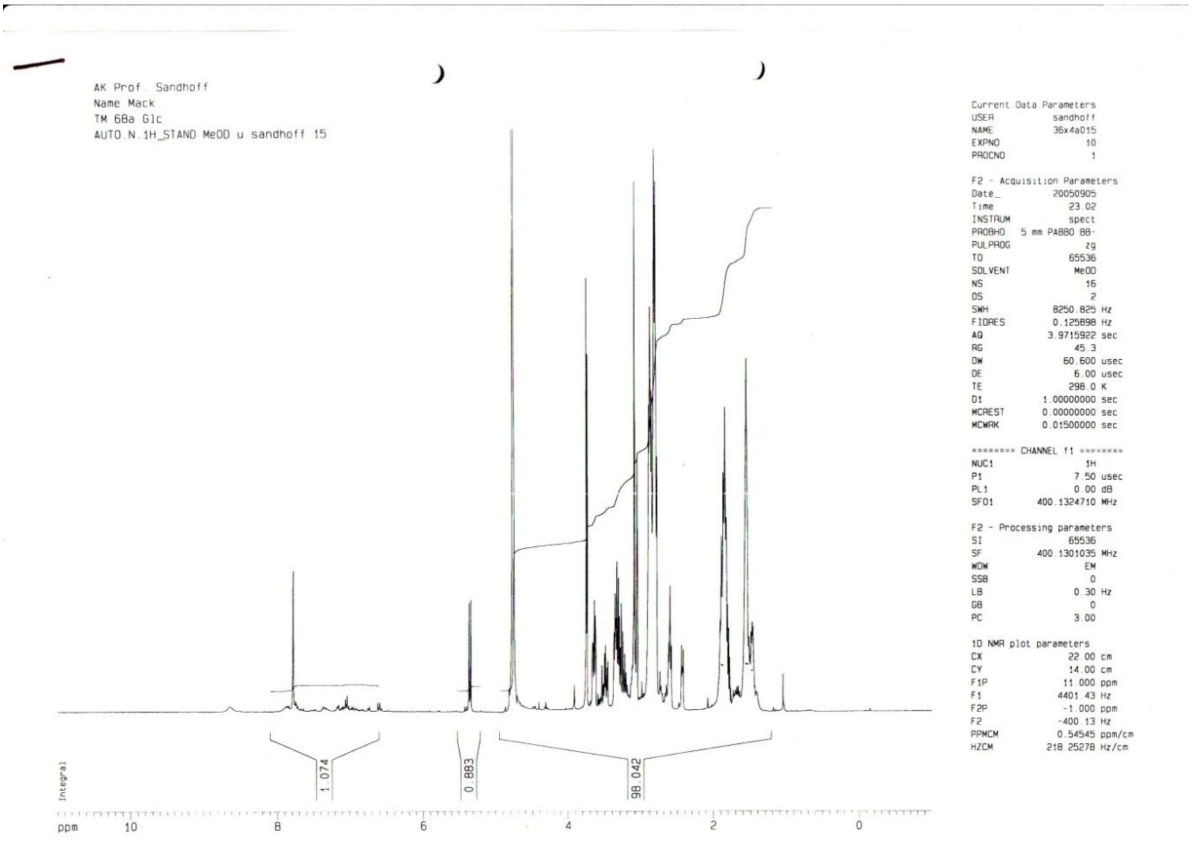

^{13}C NMR of compound 13:

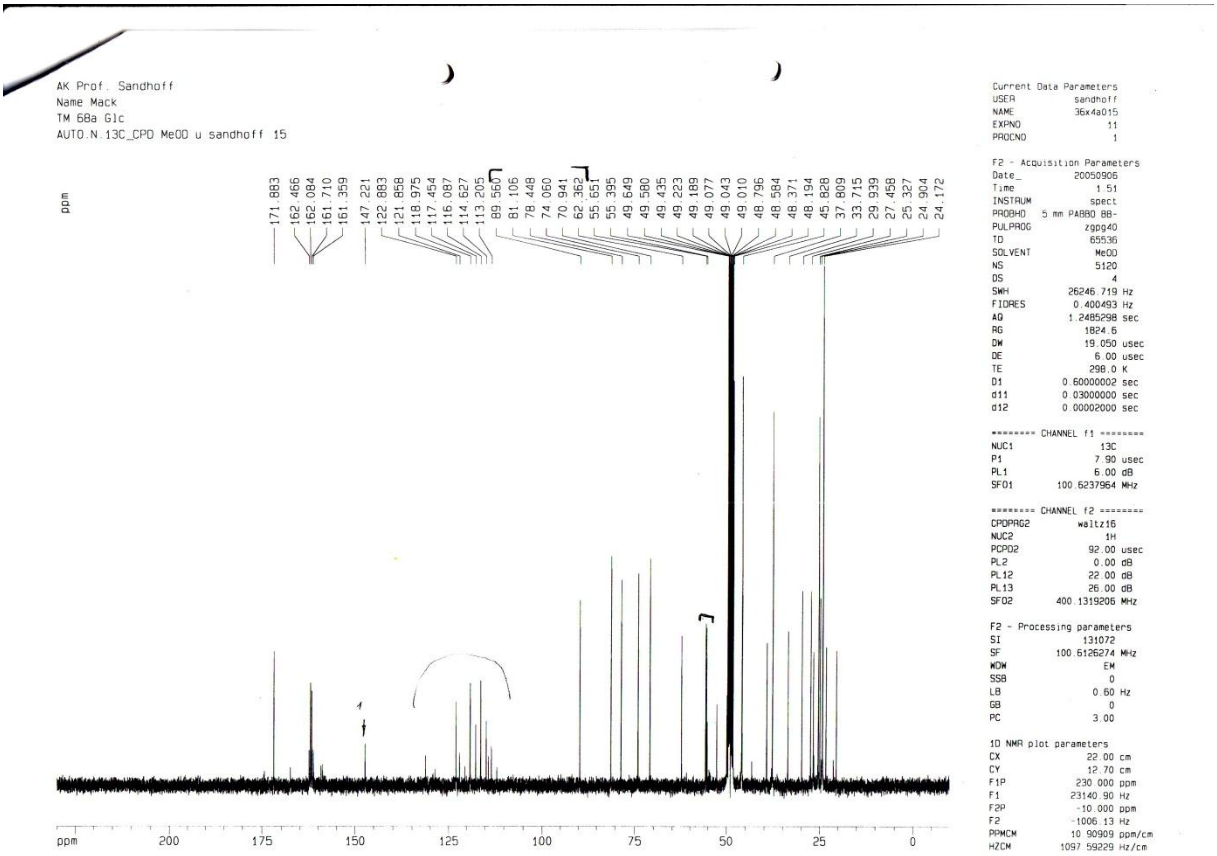

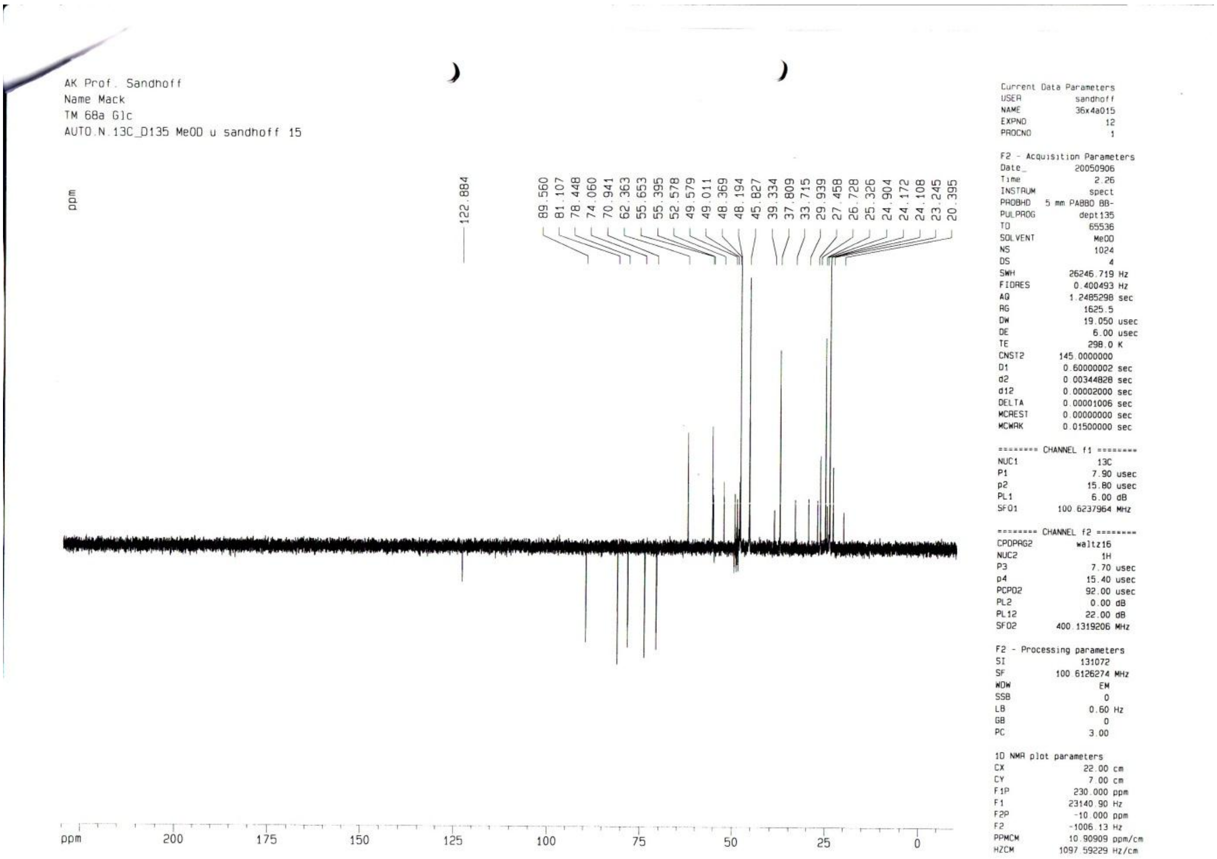

^{1}H NMR of compound 14:

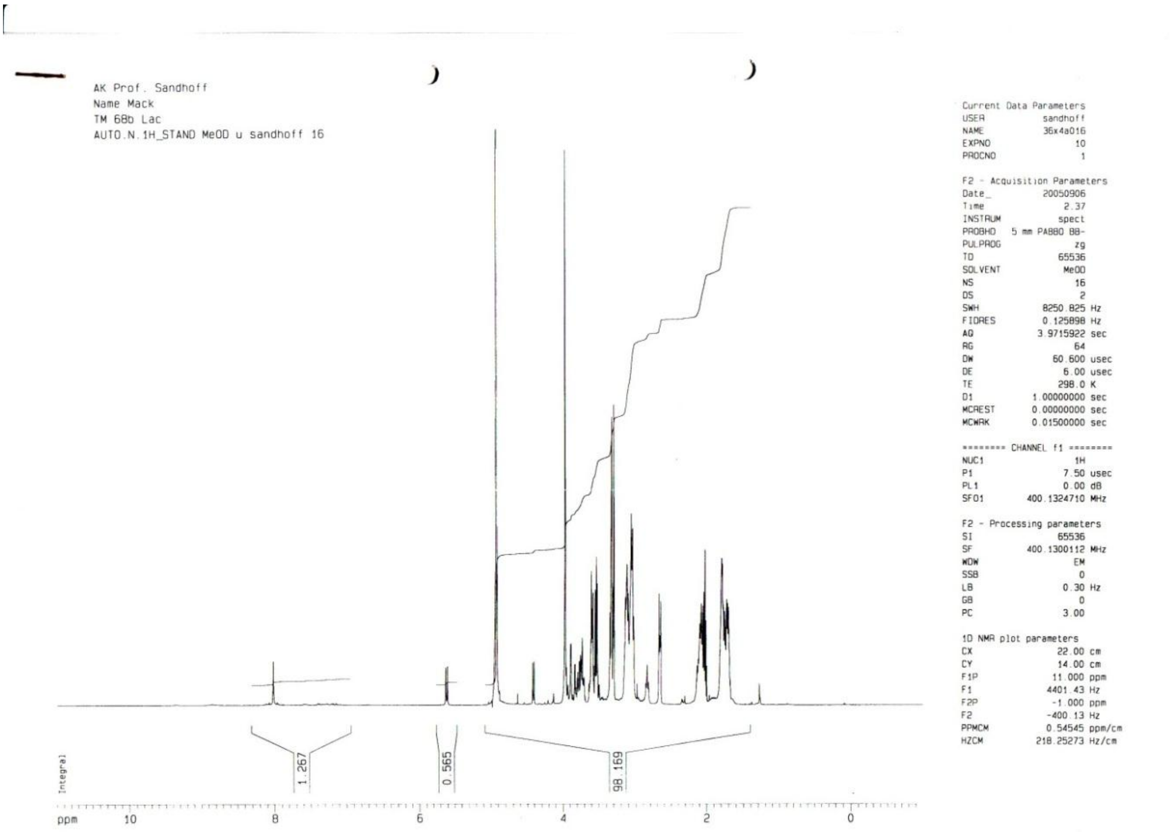

^{13}C NMR of compound 14

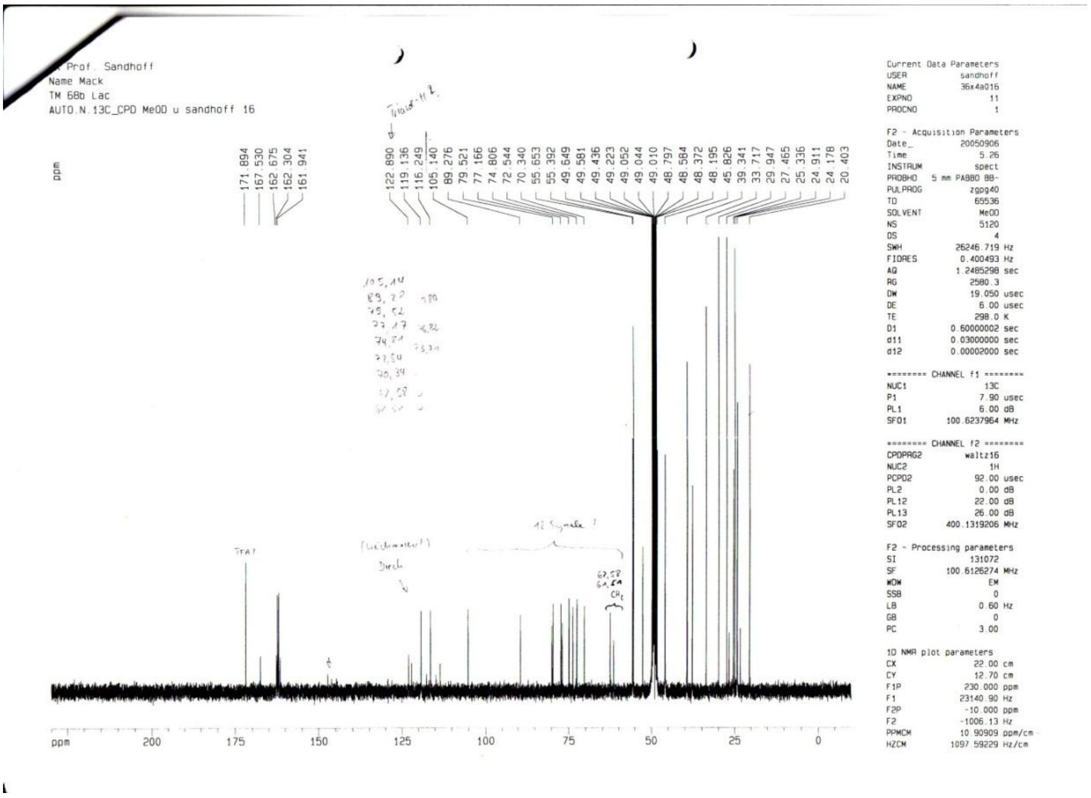

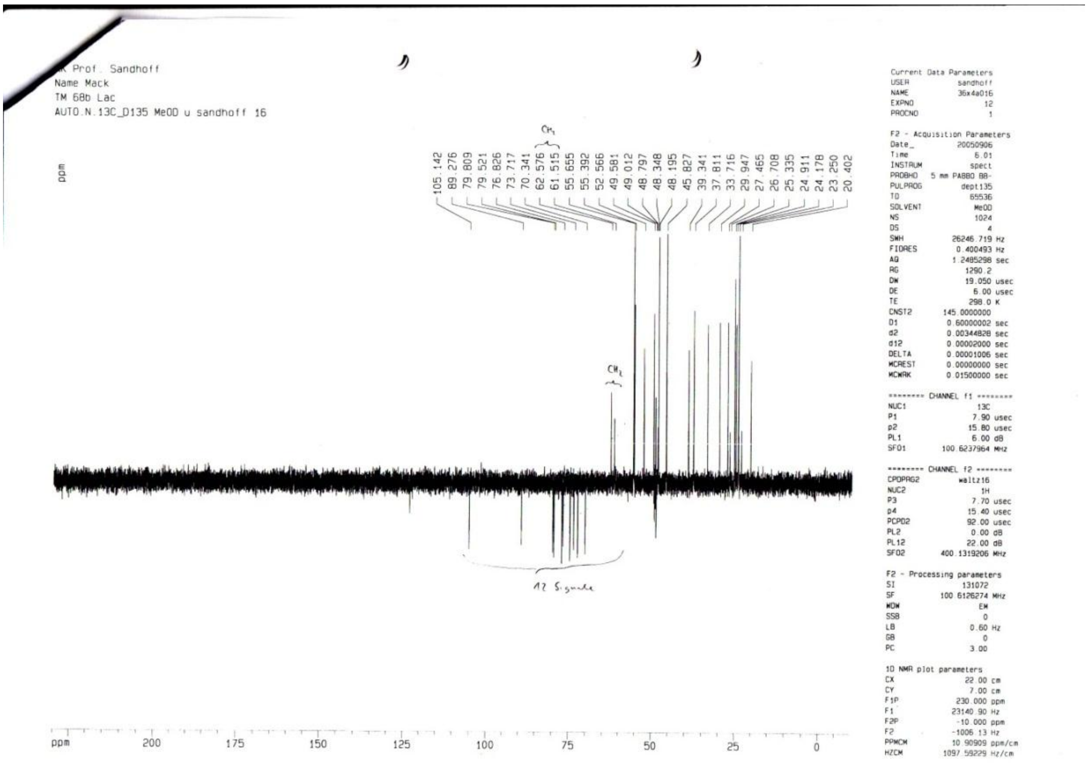

2D- NMR of compound 14

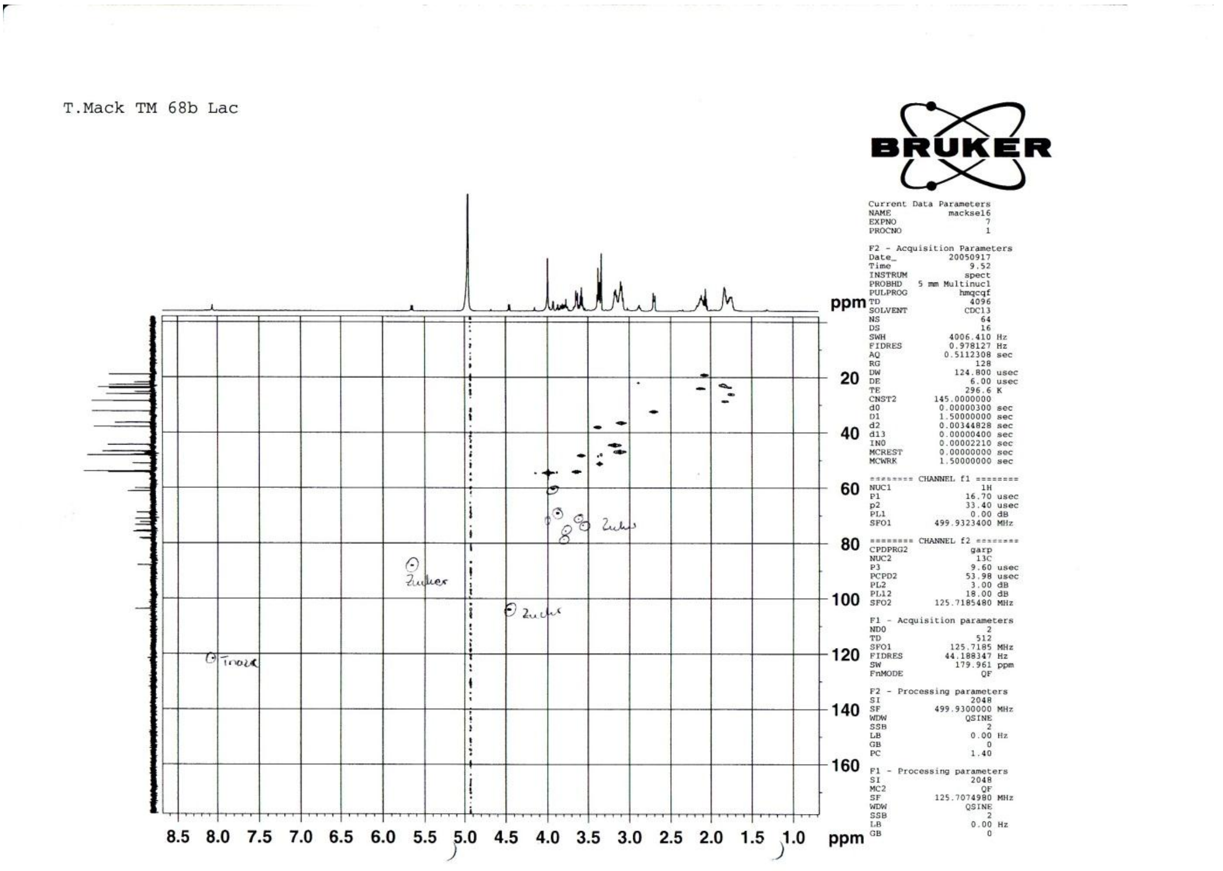

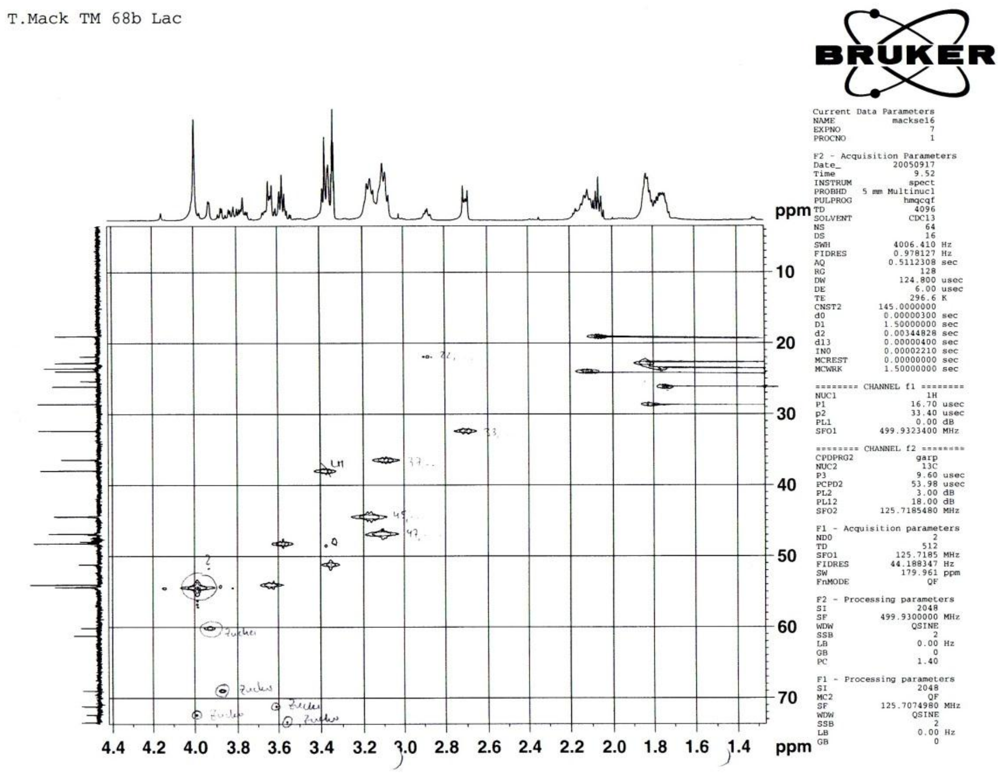
T.Mack TM 68b Lac
BRUKER
Current Data Parameters
NAME mackse16
EXPNO 7
PROCNO 1
F2 - Acquisition Parameters
Date_ 20050917
Time 9.52
INSTRUM spect
PROBHD 5 mm Multinucl
PULPROG hmqcgf
TD 4096
SOLVENT CDCl3
NS 64
DS 16
SWH 4006.410 Hz
FIDRES 0.978127 Hz
AQ 0.5112308 sec
RG 128
DW 124.800 usec
DE 6.00 usec
TE 296.6 K
CNST2 145.0000000
d0 0.00000300 sec
D1 1.50000000 sec
d2 0.00344828 sec
d13 0.00000400 sec
IN0 0.00002210 sec
MCREST 0.00000000 sec
MCWRK 1.50000000 sec
======== CHANNEL f1 ========
NUC1 1H
P1 16.70 usec
p2 33.40 usec
PL1 0.00 dB
SFO1 499.9323400 MHz
======== CHANNEL f2 ========
CPDPRG2 garp
NUC2 13C
P3 9.60 usec
PCPD2 53.98 usec
PL2 3.00 dB
PL12 18.00 dB
SFO2 125.7185480 MHz
F1 - Acquisition parameters
ND0 2
TD 512
SFO1 125.7185 MHz
FIDRES 44.188347 Hz
SW 179.961 ppm
FnMODE QF
F2 - Processing parameters
SI 2048
SF 499.9300000 MHz
WDW QSINE
SSB 2
LB 0.00 Hz
GB 0
PC 1.40
F1 - Processing parameters
SI 2048
MC2 QF
SF 125.7074980 MHz
WDW QSINE
SSB 2
LB 0.00 Hz
GB 0
ppm
10
20
30
40
50
60
70
4.4 4.2 4.0 3.8 3.6 3.4 3.2 3.0 2.8 2.6 2.4 2.2 2.0 1.8 1.6 1.4 ppm

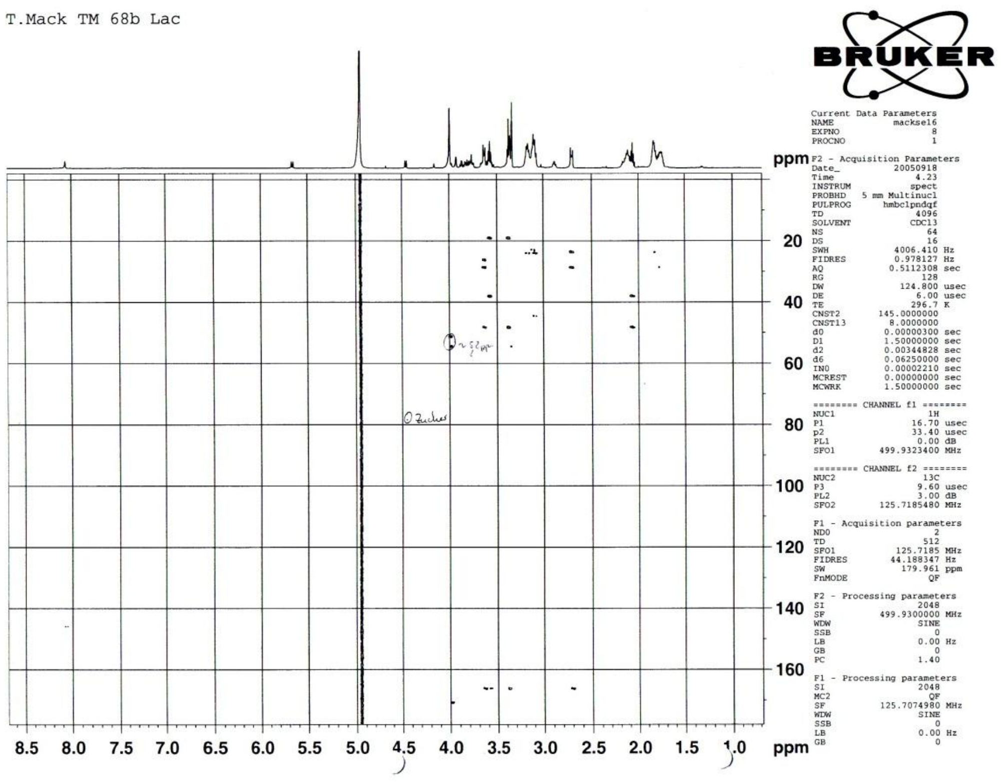
T.Mack TM 68b Lac
BRUKER
Current Data Parameters
NAME mackse16
EXPNO 8
PROCNO 1
F2 - Acquisition Parameters
Date_ 20050918
Time 4.23
INSTRUM spect
PROBHD 5 mm Multinucl
PULPROG hmbclpndqf
TD 4096
SOLVENT CDCl3
NS 64
DS 16
SWH 4006.410 Hz
FIDRES 0.978127 Hz
AQ 0.5112308 sec
RG 128
DW 124.800 usec
DE 6.00 usec
TE 296.7 K
CNST2 145.0000000
CNST13 8.0000000
d0 0.00000300 sec
D1 1.50000000 sec
d2 0.00344828 sec
d6 0.06250000 sec
IN0 0.00002210 sec
MCREST 0.00000000 sec
MCWRK 1.50000000 sec
======== CHANNEL f1 ========
NUC1 1H
P1 16.70 usec
p2 33.40 usec
PL1 0.00 dB
SFO1 499.9323400 MHz
======== CHANNEL f2 ========
NUC2 13C
P3 9.60 usec
PL2 3.00 dB
SFO2 125.7185480 MHz
F1 - Acquisition parameters
ND0 2
TD 512
SFO1 125.7185 MHz
FIDRES 44.188347 Hz
SW 179.961 ppm
FnMODE QF
F2 - Processing parameters
SI 2048
SF 499.9300000 MHz
WDW SINE
SSB 0
LB 0.00 Hz
GB 0
PC 1.40
F1 - Processing parameters
SI 2048
MC2 QF
SF 125.7074980 MHz
WDW SINE
SSB 0
LB 0.00 Hz
GB 0
ppm
20
40
60
80
100
120
140
160
8.5 8.0 7.5 7.0 6.5 6.0 5.5 5.0 4.5 4.0 3.5 3.0 2.5 2.0 1.5 1.0 ppm

Compound **15**:

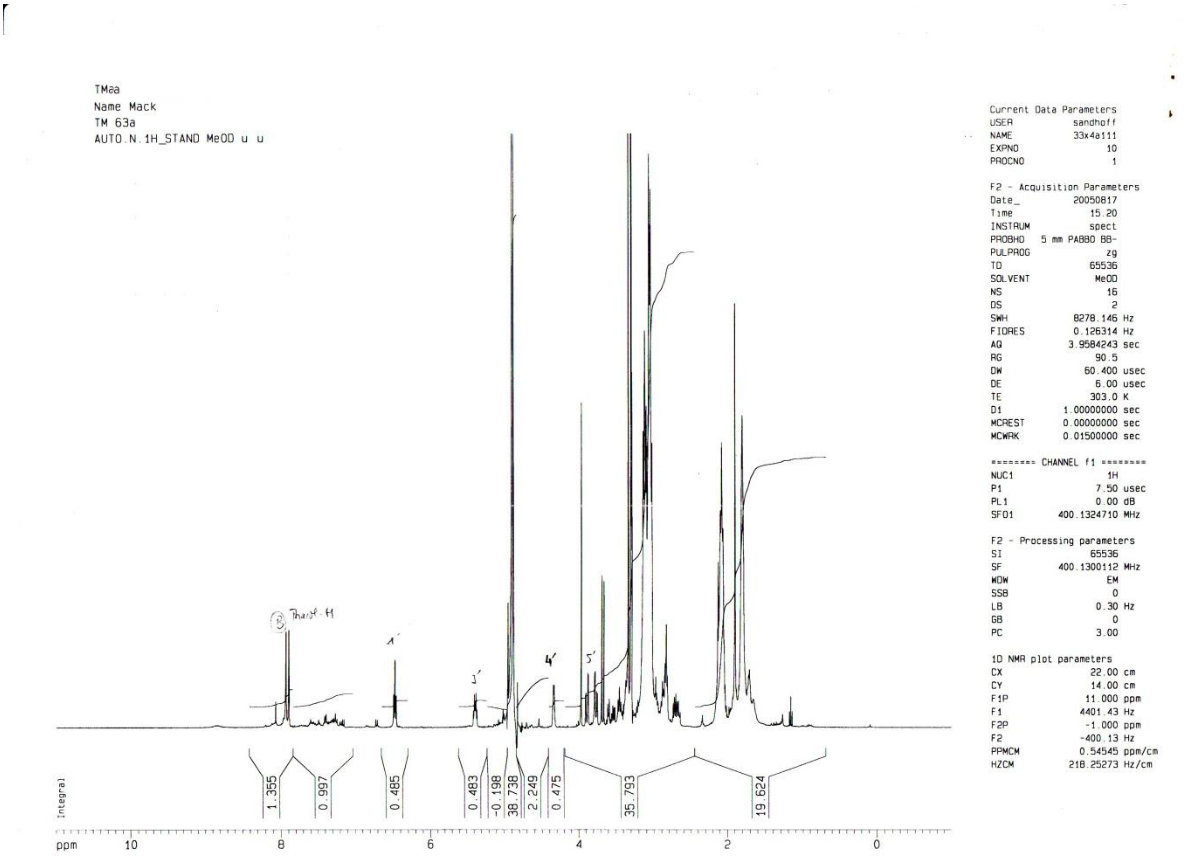
TMaa
Name Mack
TM 63a
AUTO.N.1H_STAND MeOD u u
Current Data Parameters
USER sandhoff
NAME 33x4a111
EXPNO 10
PROCNO 1
F2 - Acquisition Parameters
Date_ 20050817
Time 15.20
INSTRUM spect
PROBHD 5 mm PABBO BB-
PULPROG zg
TD 65536
SOLVENT MeOD
NS 16
DS 2
SWH 8278.146 Hz
FIDRES 0.126314 Hz
AQ 3.9584243 sec
RG 90.5
DW 60.400 usec
DE 6.00 usec
TE 303.0 K
D1 1.00000000 sec
MCREST 0.00000000 sec
MCWRK 0.01500000 sec
======== CHANNEL f1 ========
NUC1 1H
P1 7.50 usec
PL1 0.00 dB
SFO1 400.1324710 MHz
F2 - Processing parameters
SI 65536
SF 400.1300112 MHz
WDW EM
SSB 0
LB 0.30 Hz
GB 0
PC 3.00
1D NMR plot parameters
CX 22.00 cm
CY 14.00 cm
F1P 11.000 ppm
F1 4401.43 Hz
F2P -1.000 ppm
F2 -400.13 Hz
PPMCM 0.54545 ppm/cm
HZCM 218.25273 Hz/cm
1'
3'
4'
5'
Integral
1.355
0.997
0.485
0.483
-0.198
38.738
2.249
0.475
35.793
19.624
ppm
10
8
6
4
2
0

Compound **21**, **24** and **28**:

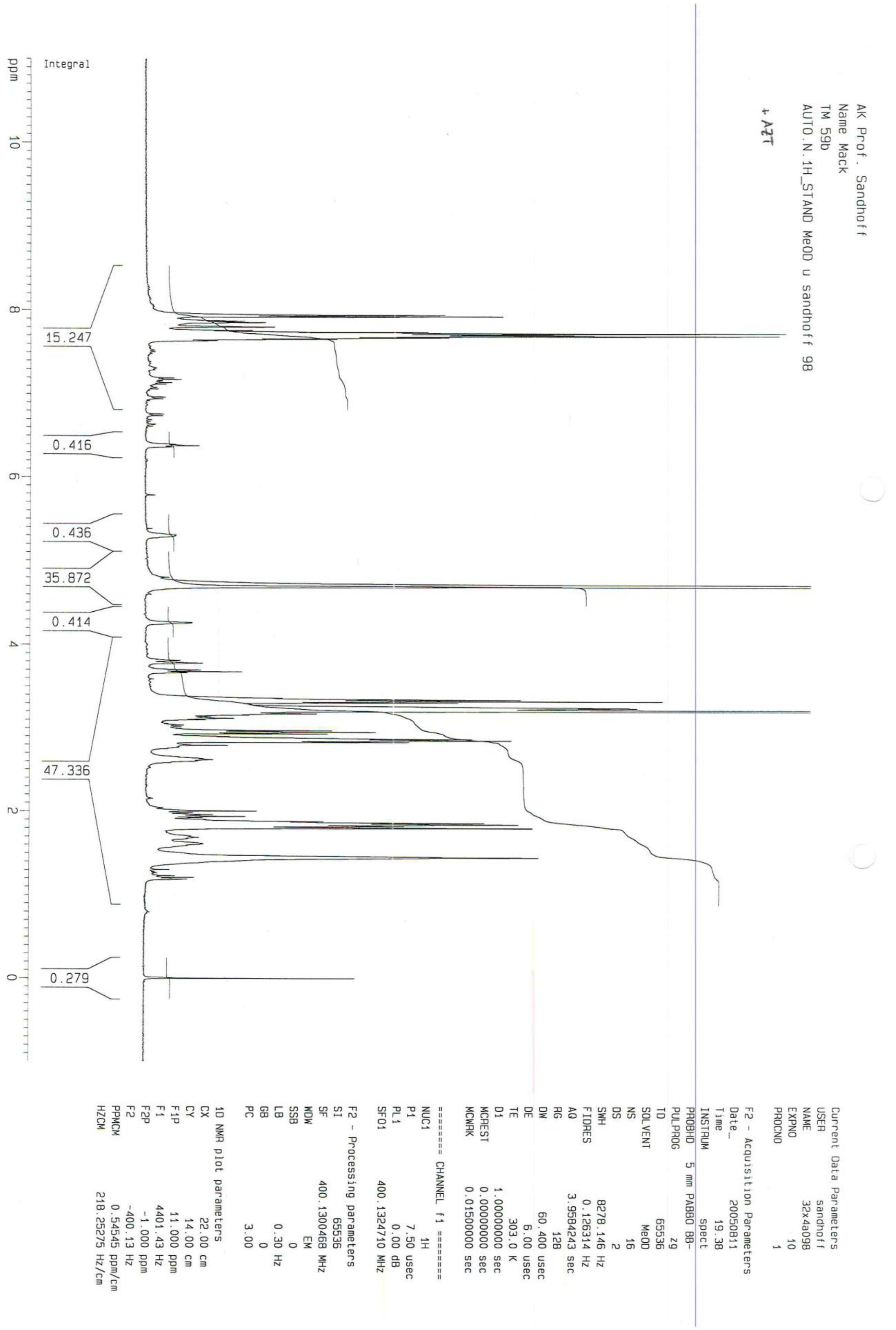

AK Prof. Sandhoff
Name Mack
TM 59b
AUTO.N.13C_CPD MeOD u sandhoff 98

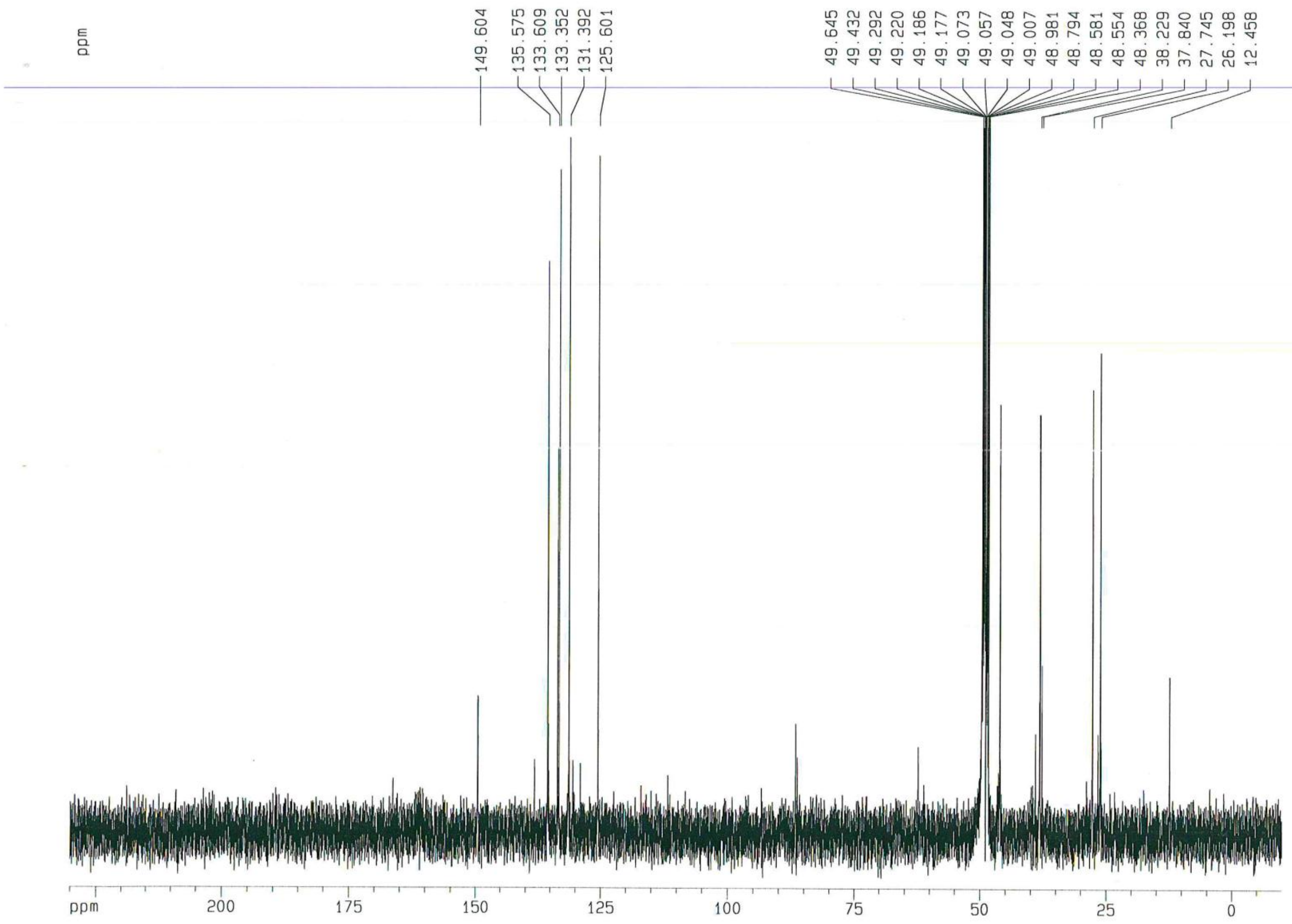

Current Data Parameters

USER	sandhoff
NAME	32x4a098
EXPNO	11
PROCNO	1

F2 - Acquisition Parameters

Date_	20050811
Time	22.27
INSTRUM	spect
PROBHD	5 mm PABBO BB-
PULPROG	zgpg40
TD	65536
SOLVENT	MeOD
NS	5120
DS	4
SWH	26178.010 Hz
FIDRES	0.399445 Hz
AQ	1.2517875 sec
RG	5160.6
DW	19.100 usec
DE	6.00 usec
TE	303.0 K
D1	0.60000002 sec
d11	0.03000000 sec
d12	0.00002000 sec

======== CHANNEL f1 ========

NUC1	13C
P1	7.90 usec
PL1	6.00 dB
SFO1	100.6237964 MHz

======== CHANNEL f2 ========

CPDPRG2	waltz16
NUC2	1H
PCPD2	92.00 usec
PL2	0.00 dB
PL12	22.00 dB
PL13	26.00 dB
SFO2	400.1319206 MHz

F2 - Processing parameters

SI	131072
SF	100.6126274 MHz
WDW	EM
SSB	0
LB	0.60 Hz
GB	0
PC	3.00

1D NMR plot parameters

CX	22.00 cm
CY	12.70 cm
F1P	230.000 ppm
F1	23140.90 Hz
F2P	-10.000 ppm
F2	-1006.13 Hz
PPMCM	10.90909 ppm/cm
HZCM	1097.59229 Hz/cm

AK Prof. Sandhoff
Name Mack
TM 59b
AUTO.N.13C_D135 MeOD u sandhoff 98

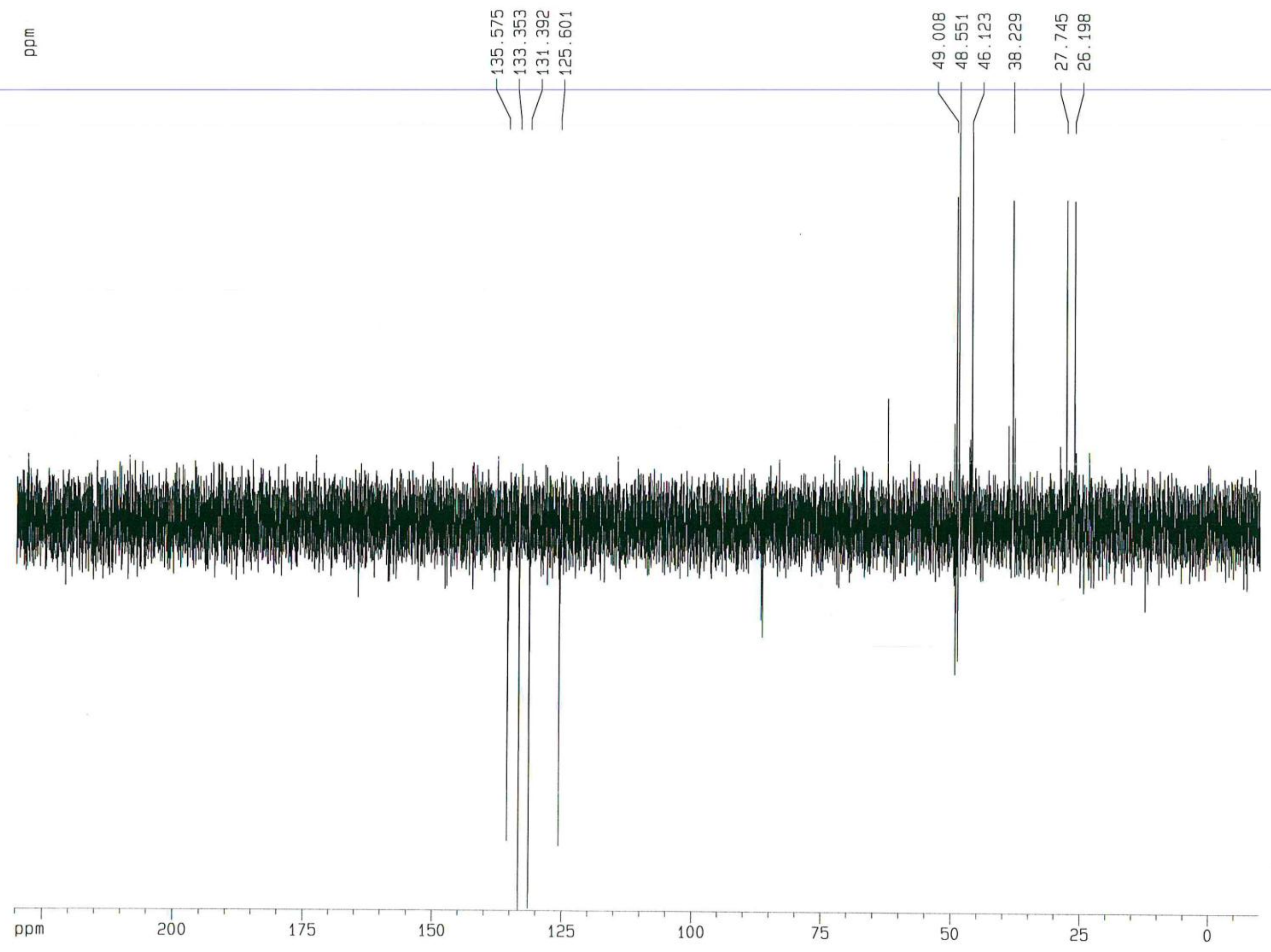

Current Data Parameters

USER	sandhoff
NAME	32x4a098
EXPNO	12
PROCNO	1

F2 - Acquisition Parameters

Date_	20050811
Time	23.02
INSTRUM	spect
PROBHD	5 mm PABBO BB-
PULPROG	dept135
TD	65536
SOLVENT	MeOD
NS	1024
DS	4
SWH	26178.010 Hz
FIDRES	0.399445 Hz
AQ	1.2517875 sec
RG	20642.5
DW	19.100 usec
DE	6.00 usec
TE	303.0 K
CNST2	145.0000000
D1	0.60000002 sec
d2	0.00344828 sec
d12	0.00002000 sec
DELTA	0.00001006 sec
MCREST	0.00000000 sec
MCWRK	0.01500000 sec

======== CHANNEL f1 ========

NUC1	13C
P1	7.90 usec
p2	15.80 usec
PL1	6.00 dB
SFO1	100.6237964 MHz

======== CHANNEL f2 ========

CPDPRG2	waltz16
NUC2	1H
P3	7.70 usec
p4	15.40 usec
PCPD2	92.00 usec
PL2	0.00 dB
PL12	22.00 dB
SFO2	400.1319206 MHz

F2 - Processing parameters

SI	131072
SF	100.6126274 MHz
WDW	EM
SSB	0
LB	0.60 Hz
GB	0
PC	3.00

1D NMR plot parameters

CX	22.00 cm
CY	7.00 cm
F1P	230.000 ppm
F1	23140.90 Hz
F2P	-10.000 ppm
F2	-1006.13 Hz
PPMCM	10.90909 ppm/cm
HZCM	1097.59229 Hz/cm

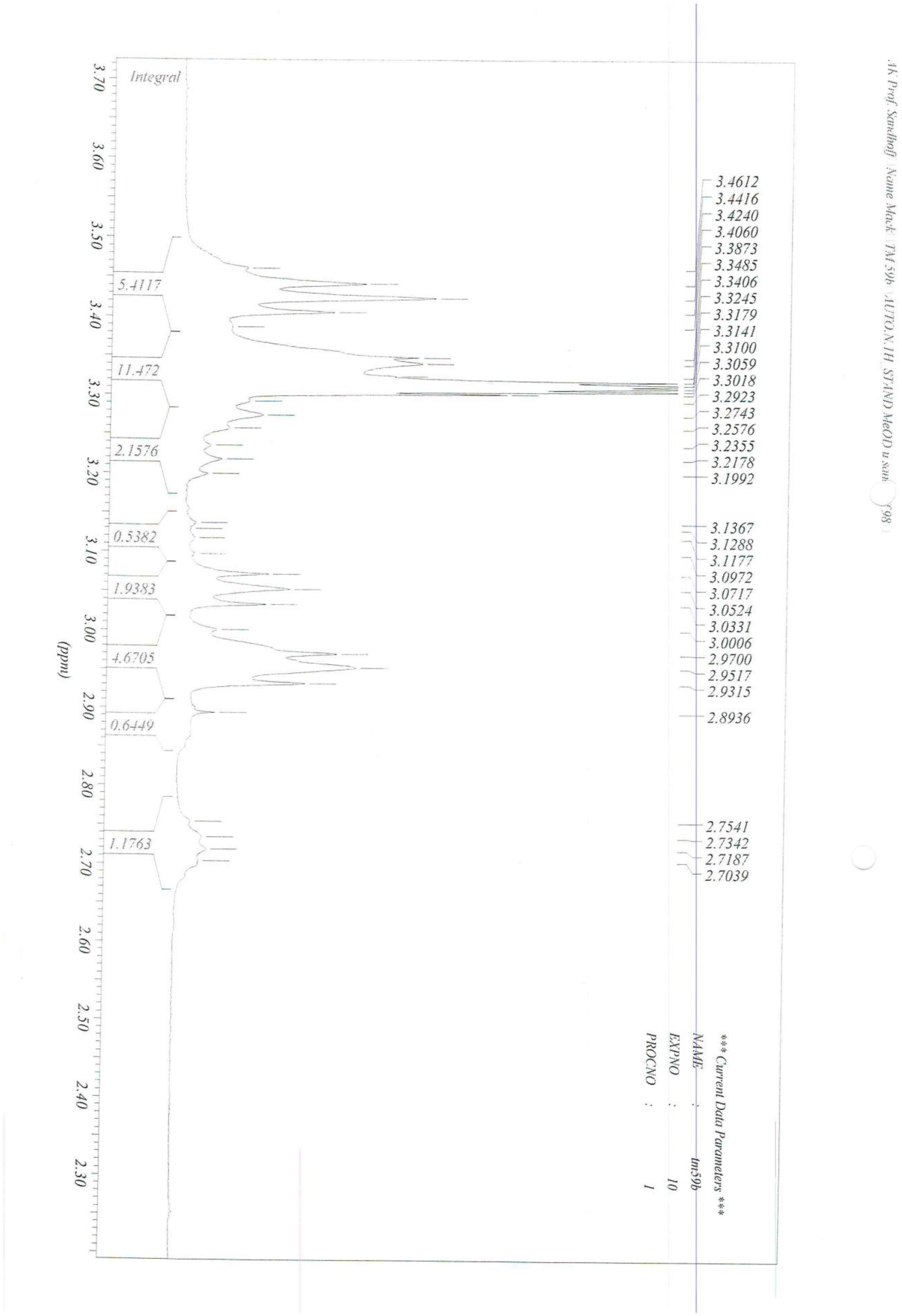
AK Prof. Sandhoff Name Mack TM 59b AUTO.N.1H STAND MeOD u sam
Integral
5.4117
11.472
2.1576
0.5382
1.9383
4.6705
0.6449
1.1763
3.4612
3.4416
3.4240
3.4060
3.3873
3.3485
3.3406
3.3245
3.3179
3.3141
3.3100
3.3059
3.3018
3.2923
3.2743
3.2576
3.2355
3.2178
3.1992
3.1367
3.1288
3.1177
3.0972
3.0717
3.0524
3.0331
3.0006
2.9700
2.9517
2.9315
2.8936
2.7541
2.7342
2.7187
2.7039
*** Current Data Parameters ***
NAME : tm59b
EXPNO : 10
PROCNO : 1
(ppm)
3.70
3.60
3.50
3.40
3.30
3.20
3.10
3.00
2.90
2.80
2.70
2.60
2.50
2.40
2.30

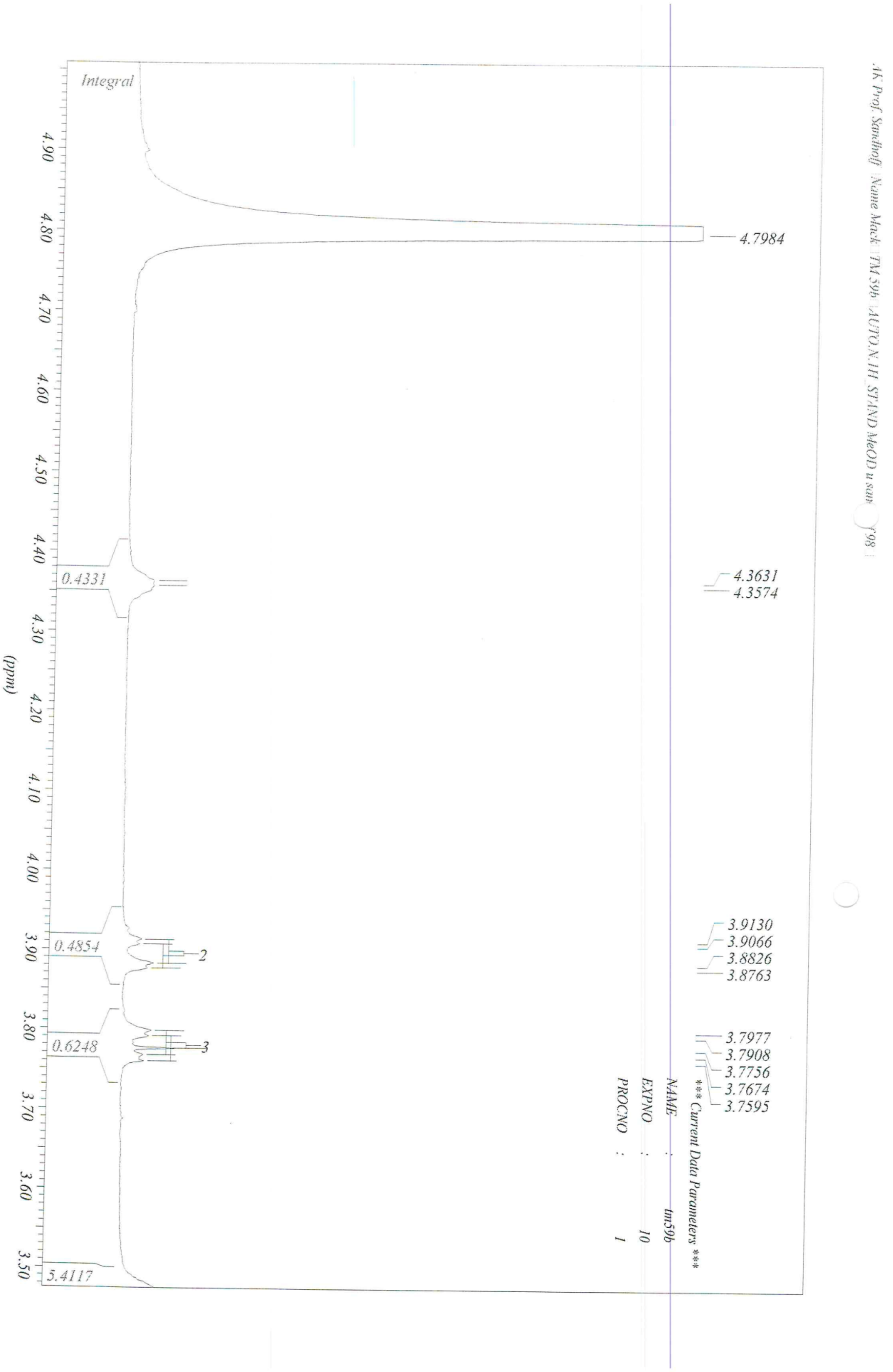
AK Prof. Sandhoff Name Mack TM 59b AUTO.N.1H STAND MeOD u sam
Integral
4.7984
4.3631
4.3574
3.9130
3.9066
3.8826
3.8763
3.7977
3.7908
3.7756
3.7674
3.7595
0.4331
0.4854
0.6248
5.4117
2
3
*** Current Data Parameters ***
NAME : tm59b
EXPNO : 10
PROCNO : 1
4.90
4.80
4.70
4.60
4.50
4.40
4.30
4.20
4.10
4.00
3.90
3.80
3.70
3.60
3.50
(ppm)

AK Prof. Sandhoff Name Mack TM 59b AUTO.N.1H_STAND MeOD u sam[illegible] [illegible]98

*** Current Data Parameters ***

NAME : tm59b
EXPNO : 10
PROCNO : 1

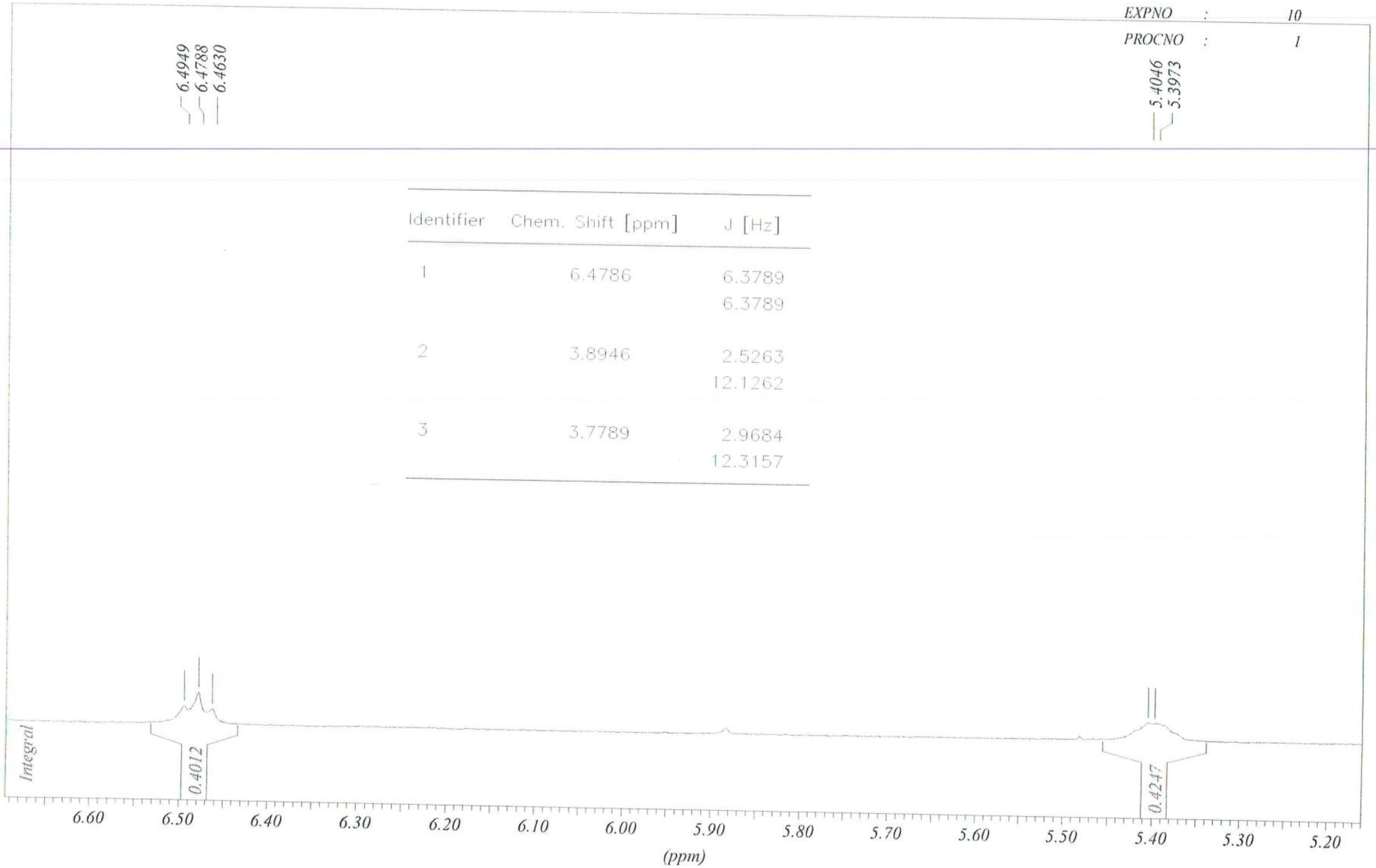

Identifier	Chem. Shift [ppm]	J [Hz]
1	6.4786	6.3789<br>6.3789
2	3.8946	2.5263<br>12.1262
3	3.7789	2.9684<br>12.3157

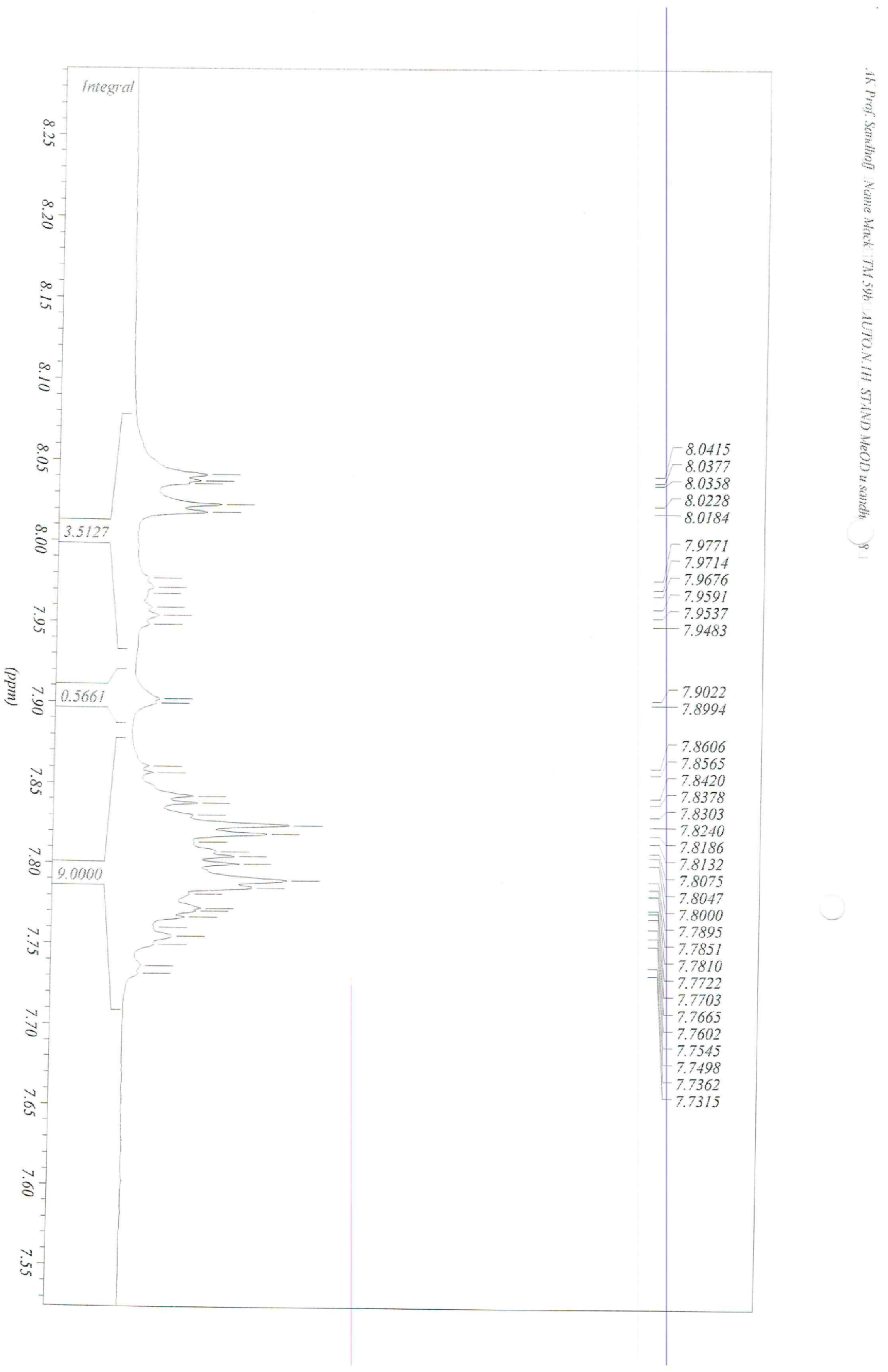
AK Prof. Sandhoff Name Mack TM 59b AUTO.N 1H_STAND MeOD u sandh
Integral
3.5127
0.5661
9.0000
(ppm)
8.25
8.20
8.15
8.10
8.05
8.00
7.95
7.90
7.85
7.80
7.75
7.70
7.65
7.60
7.55
8.0415
8.0377
8.0358
8.0228
8.0184
7.9771
7.9714
7.9676
7.9591
7.9537
7.9483
7.9022
7.8994
7.8606
7.8565
7.8420
7.8378
7.8303
7.8240
7.8186
7.8132
7.8075
7.8047
7.8000
7.7895
7.7851
7.7810
7.7722
7.7703
7.7665
7.7602
7.7545
7.7498
7.7362
7.7315

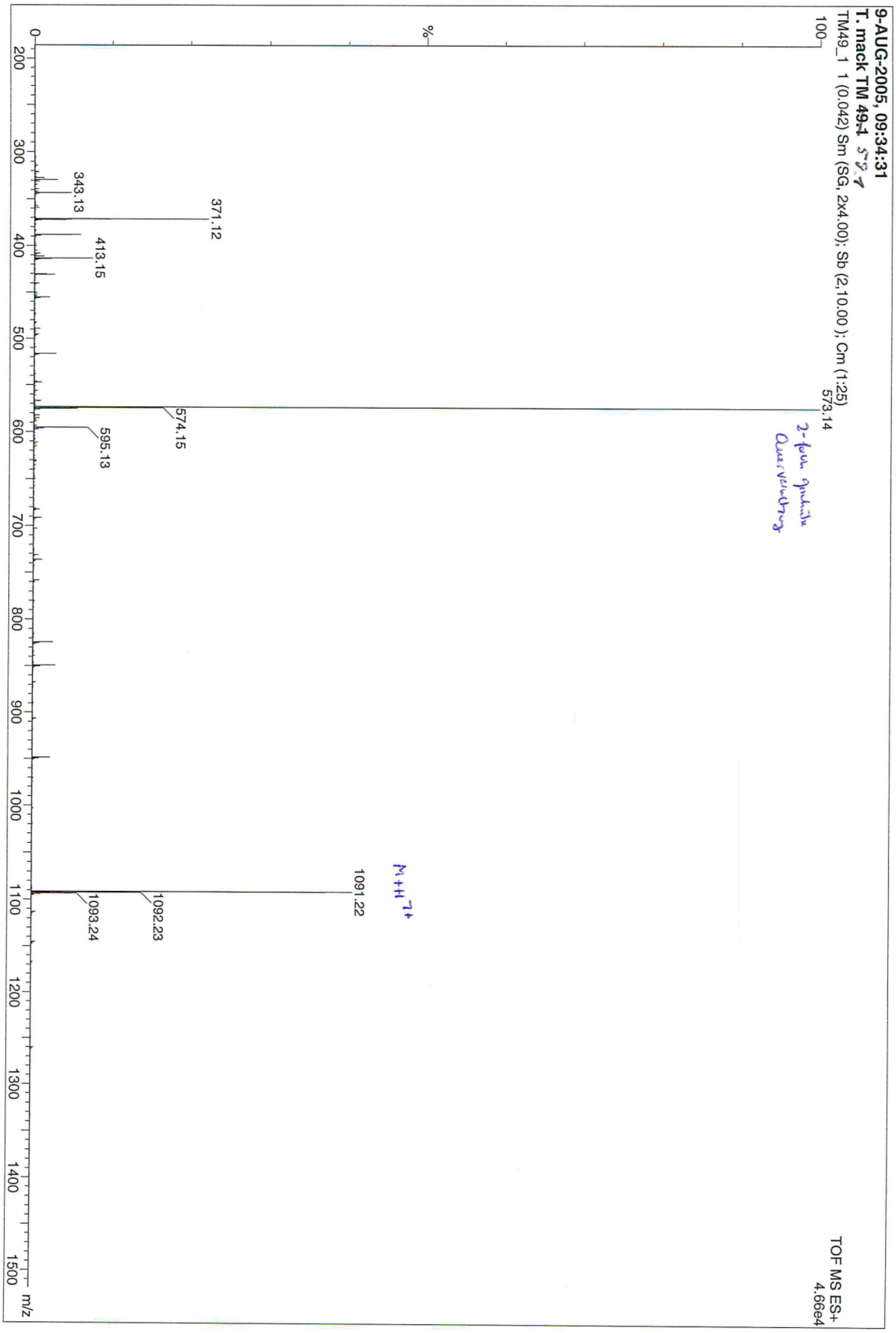
9-AUG-2005, 09:34:31
TM49_1 1 (0.042) Sm (SG, 2x4.00); Sb (2,10.00); Cm (1:25)
TOF MS ES+
4.66e4
100
%
0
573.14
371.12
343.13
413.15
574.15
595.13
1091.22
1092.23
1093.24
200
300
400
500
600
700
800
900
1000
1100
1200
1300
1400
1500
m/z

AK Prof. Sandhoff
Name Mack
TM 59a
AUTO.N.1H_STAND DMSO u sandhoff 20

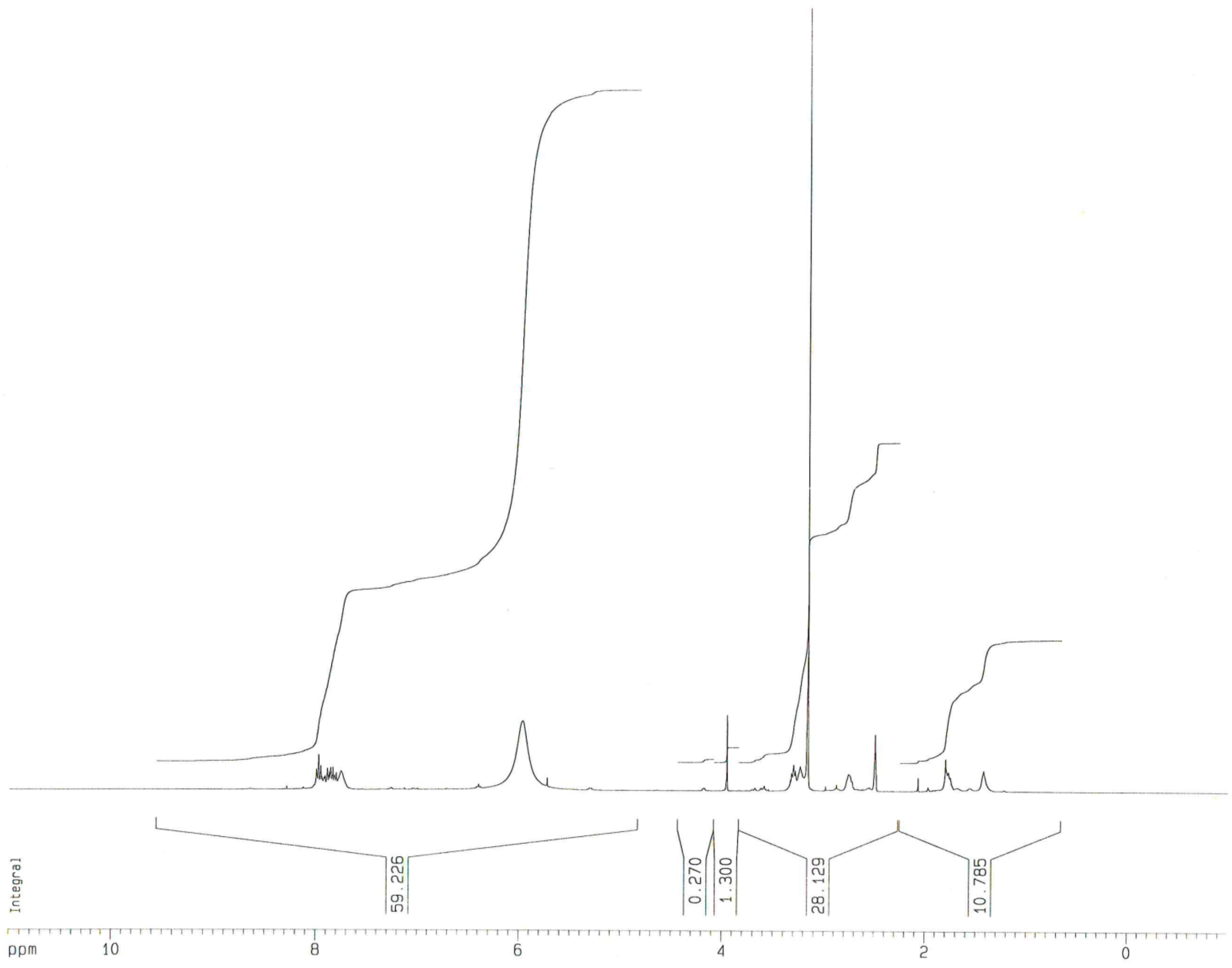

Current Data Parameters

USER	sandhoff
NAME	32x4a020
EXPNO	10
PROCNO	1

F2 - Acquisition Parameters

Date_	20050808
Time	16.25
INSTRUM	spect
PROBHD	5 mm PABBO BB-
PULPROG	zg
TD	65536
SOLVENT	DMSO
NS	16
DS	2
SWH	8278.146 Hz
FIDRES	0.126314 Hz
AQ	3.9584243 sec
RG	71.8
DW	60.400 usec
DE	6.00 usec
TE	303.0 K
D1	1.00000000 sec
MCREST	0.00000000 sec
MCWRK	0.01500000 sec

======== CHANNEL f1 ========

NUC1	1H
P1	7.50 usec
PL1	0.00 dB
SFO1	400.1324710 MHz

F2 - Processing parameters

SI	65536
SF	400.1300060 MHz
WDW	EM
SSB	0
LB	0.30 Hz
GB	0
PC	3.00

1D NMR plot parameters

CX	22.00 cm
CY	14.00 cm
F1P	11.000 ppm
F1	4401.43 Hz
F2P	-1.000 ppm
F2	-400.13 Hz
PPMCM	0.54545 ppm/cm
HZCM	218.25273 Hz/cm

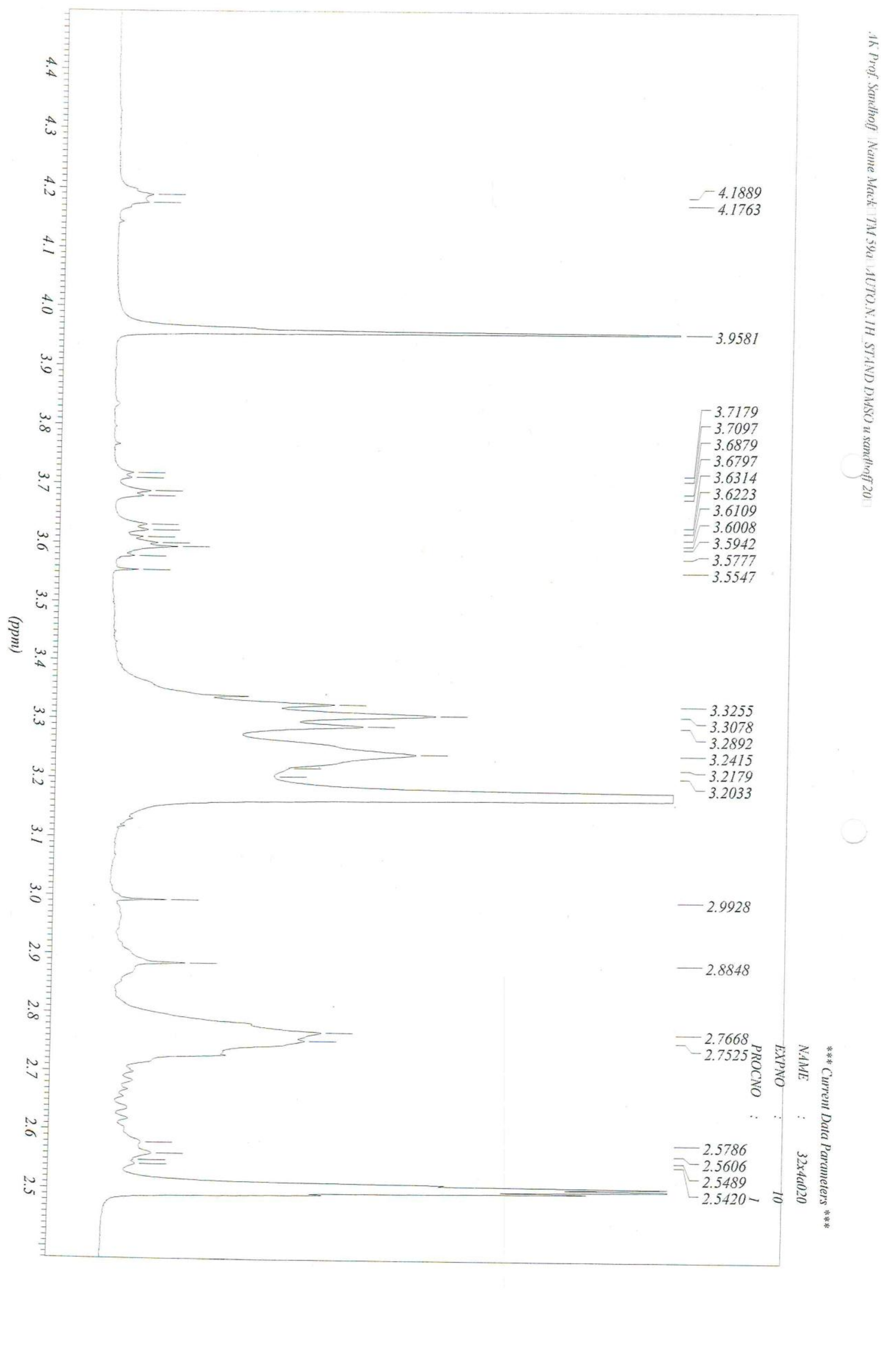
4.1889
4.1763
3.9581
3.7179
3.7097
3.6879
3.6797
3.6314
3.6223
3.6109
3.6008
3.5942
3.5777
3.5547
3.3255
3.3078
3.2892
3.2415
3.2179
3.2033
2.9928
2.8848
2.7668
2.7525
2.5786
2.5606
2.5489
2.5420
4.4
4.3
4.2
4.1
4.0
3.9
3.8
3.7
3.6
3.5
3.4
3.3
3.2
3.1
3.0
2.9
2.8
2.7
2.6
2.5
(ppm)
*** Current Data Parameters ***
NAME : 32x4a020
EXPNO : 10
PROCNO : 1

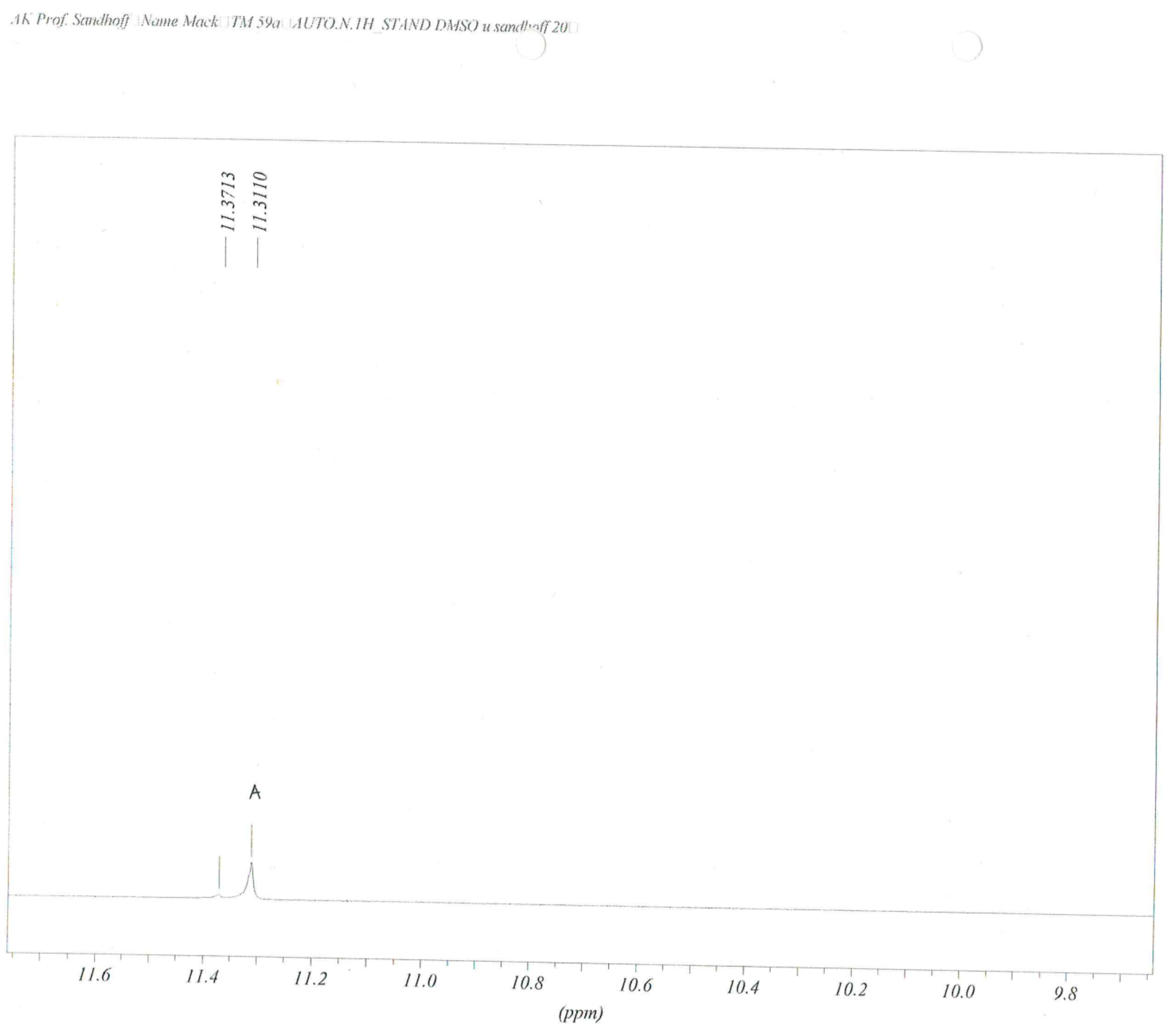

*** Current Data Parameters ***

NAME	:	32x4a020
EXPNO	:	10
PROCNO	:	1
